# Co_3_O_4_/C‐NFs Induced 3D Electric Field Enhancement for Dual‐Regulation of Polysulfides and Li^+^ Transport in Lithium–Sulfur Batteries

**DOI:** 10.1002/advs.202520167

**Published:** 2025-11-25

**Authors:** Zhijie Qi, Jun Jiang, Pengcheng Yao, Zhenjie Lu, Ying Shen, Shujun Liu, Jingwen Sun, Pan Xiong, Xin Wang, Xiaoping Ouyang, Junwu Zhu, Yongsheng Fu

**Affiliations:** ^1^ Key Laboratory for Soft Chemistry and Functional Materials School of Chemistry and Chemical Engineering Nanjing University of Science and Technology Nanjing 210094 China; ^2^ Department of Chemical and Biomolecular Engineering and Ralph O'Connor Sustainable Energy Institute Johns Hopkins University Baltimore Maryland 21218 USA; ^3^ Key Laboratory of Low Dimensional Materials and Application Technology School of Materials Science and Engineering Xiangtan University Xiangtan 411105 China

**Keywords:** Co_3_O_4_ nano‐catalysts, in situ electrochemical technique, nano‐framework, sulfur reduction reaction

## Abstract

Lithium–sulfur batteries (LSBs) are promising next‐generation energy storage systems, yet their practical application is hindered by sluggish and uncontrolled mass transport of Li^+^ and lithium polysulfides (LiPSs). Here, a Co_3_O_4_‐based nano‐framework catalyst (Co_3_O_4_/C‐NFs) is designed featuring a highly symmetric 3D sharp‐edged architecture that induces strong 3D electric field enhancement. This unique structural design promotes the directional migration and enrichment of negatively charged species, thereby accelerating the sulfur reduction reaction (SRR) kinetics, suppressing LiPSs shuttling, and improving Li⁺ transport. Moreover, by monitoring the real‐time conversion of LiPSs during the reaction using integrated in situ techniques, a universal method for quantitatively evaluating LiPSs conversion kinetics is proposed. Both experimental and theoretical results confirm that the 3D electric field enhancement governs mass transfer at multiple scales, achieving efficient ion regulation and interfacial stability. Benefiting from this effect, the assembled double‐layer pouch cell with a high sulfur loading of 6.5 mg cm^−2^ and a lean electrolyte ratio (E/S = 4 µL mg^−1^) maintains stable cycling for over 100 cycles at 0.1C, delivering a specific capacity of 1200 mAh g^−1^. This work introduces a new paradigm of 3D electric field–driven mass transfer modulation for designing high‐performance and practical LSBs.

## Graphical abstract

A nano‐framework structured Co_3_O_4_/C catalyst with 3D electric field enhancement is designed to facilitate the mass transfer of both Li⁺ and lithium polysulfides (LiPSs) in lithium–sulfur batteries (LSBs). The enhanced electric‐field–driven migration significantly accelerates sulfur reduction reaction (SRR) kinetics while suppressing the shuttle effect and Li dendrite formation, thereby effectively improving the overall performance of LSBs.

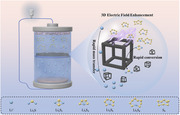

## 1 Introduction

Mass transfer, as a fundamental and essential physical phenomenon, directly impacts the reaction rate and efficiency within the systems of energy storage and conversion.^[^ ^1^, ^2^, ^3^ ^]^ Its significance spans diverse scientific and industrial applications. In electrochemical energy storage systems like batteries, efficient mass transfer ensures the prompt supply of reactants to the reaction sites, which is essential for maintaining a high power output and prolonging the battery life.^[^ ^4^, ^5^ ^]^ Consequently, mass transfer serves as a key driving force for achieving breakthroughs and optimizations in the domain of energy storage and conversion.

Lithium–Sulfur batteries (LSBs) have garnered extensive attention and are broadly regarded as a highly promising candidate for the next generation of high energy density energy storage technology due to their extremely high theoretical energy density (2600 Wh kg^−1^), low cost, resource abundance, and environmental protection.^[^ ^6^, ^7^, ^8^ ^]^ Nevertheless, during the charging and discharging process, the uncontrollable mass transfer of Li^+^ and lithium polysulfides (LiPSs) becomes a major bottleneck, which severely restricts the practical application of LSBs in real‐world scenarios.^[^ ^9^, ^10^, ^11^, ^12^ ^]^ In the electrolyte, the generation of an unfavorable solvation structure of Li^+^ through the interaction with solvent molecules, substantially hampers the transfer efficiency of Li^+^, which consequently accelerates the nucleation and growth of dendrites in the anode region, compromising the battery's performance and safety.^[^ ^13^, ^14^ ^]^ Simultaneously, the shuttle effect, driven by the mass transfer of LiPSs migrating toward the anode, triggers irreversible reactions with the anode's metallic Li, resulting in active material loss, capacity decline, and decreased coulombic efficiency, and it will also affect the transport of Li^+^.^[^ ^15^, ^16^ ^]^ Additionally, the sluggish mass transfer of LiPSs occurring on the surface of catalysts directly impedes the kinetics of the sulfur reduction reaction (SRR), limiting the utilization of active materials and reducing the battery's cycle reversibility.^[^ ^17^, ^18^, ^19^ ^]^ Therefore, effectively governing the mass transfer of Li^+^ and LiPSs is crucial for the development of high‐performance LSBs.

The mass transfer of chemical species in LSBs can be described by the Planck–Nernst equation (neglecting the effect of convection):^[^ ^4^, ^20^ ^]^

(1) $$J=-D\nabla c+\frac{zec}{K_{B}T}DE$$

where *J* is the mass transfer flux, D is the diffusion coefficient of the chemical species, *z* is the valence of the ionic species, e is the elementary charge, K_B_ is the Boltzmann constant, T is the absolute temperature, c is the concentration of the species, and E is the electric field.

It is evident from the above equation that the mass transfer involves two principal modes: diffusion and migration, which are regulated by the concentration gradient (∇c) and the potential gradient (E), respectively. Currently, researchers have been enhancing the mass transfer of reactants by designing materials with porous structures and large specific surface areas, primarily by optimizing the diffusion process.^[^ ^21^, ^22^ ^]^ However, as the reaction continuously progresses and reactants are progressively depleted, this approach, which relies on ∇c to drive mass transfer, gradually loses its efficacy. In contrast, electric field‐driven migration is not influenced by reactant consumption. Moreover, the non‐contact force induced by the electric field can promote the accumulation of negatively charged species toward regions of higher electric field strength. Therefore, catalysts with an electric field enhancement effect on the materials’ surface can not only effectively enhance the mass transfer of LiPSs on the catalysts’ surface, thereby accelerating the reaction kinetics and alleviating the shuttle effect, but also facilitate the concentration of TFSI^−^ anions on the catalysts’ surface, promoting the dissociation of Li^+^ and enhancing the transfer of Li^+^ to the cathode electrode under a larger ∇c.

Previous studies have been dedicated to devising a series of needle and sheet‐like materials based on the *tip effect*, taking advantage of the significantly enhanced local electric field at the tip of materials to effectively accelerate the mass transfer rate of negatively charged reactants on the catalysts’ surface.^[^ ^23^, ^24^ ^]^ Nonetheless, the electric field enhancement effect observed in current needle‐like or sheet‐like materials is typically restricted to the tip regions when the bias is applied along a single 1D direction, while no significant enhancement can be generated in other directions.^[^ ^25^, ^26^ ^]^ Owing to such limitations to a single dimension and localized areas, the electric‐field enhancement cannot be effectively utilized throughout the entire 3D reaction space, thereby failing to sufficiently promote the overall reaction kinetics. Therefore, constructing materials with an electric field enhanced across a 3D region to accelerate the mass transfer and concentration of LiPSs and TFSI^−^ on the catalysts’ surface is crucial for improving the reaction kinetics of the SRR, strengthening the transport of Li^+^, and ultimately facilitating the development of high‐performance LSBs.

Herein, with the aim of implementing the strategy of 3D electric field enhancement, we have successfully designed and constructed a Co_3_O_4_‐based catalyst (Co_3_O_4_/C‐NFs) with a unique nano‐framework structure. The distinctive nano‐framework structure not only exposes a distinctive catalytically active surface area, but also incorporates numerous sharp angular regions with high 3D symmetry, thereby achieving electric field enhancement in a 3D region. Benefiting from this 3D electric field enhancement, the mass transfer of LiPSs is effectively improved, further promoting the SRR kinetics and effectively suppressing the shuttle effect. Meanwhile, for the first time, we develop a universal method to quantitatively assess the kinetics of LiPSs conversion through in situ Raman spectroscopy and X‐ray diffraction (XRD) measurements, which can monitor the internal catalytic reaction processes within Li–S batteries in real time. The obtained results clearly demonstrate that the Co_3_O_4_/C‐NFs exhibit significant acceleration in the transformation from Li_2_S_8_ to Li_2_S_6_ and the formation of Li_2_S. In addition, the migration rate of Li^+^ is significantly improved, effectively mitigating the formation of Li dendrites at the anode. Due to the improvement of SRR kinetics and Li^+^ transport by the Co_3_O_4_/C‐NFs, the decay rate of the assembled Li‐S cells was a mere 0.049% after 700 cycles at 3C, demonstrating exceptional cycle stability and safety. This work successfully clarifies that the distinctive 3D electric field enhancement characteristics are inherent to the nano‐framework structure within electrochemical reactions for improving LSBs performance.

## 2 Results and Discussion

**Figure 1:**
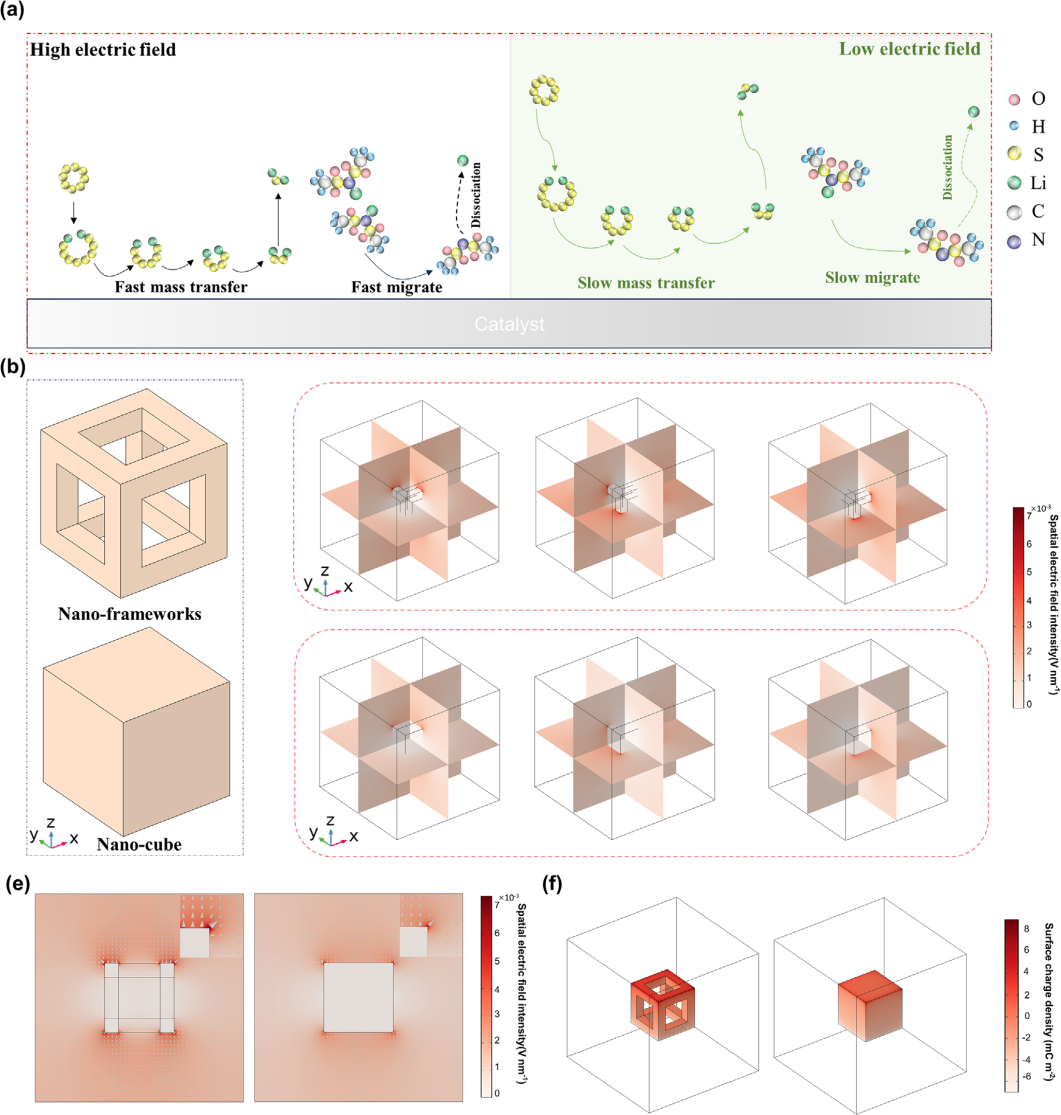
a) Schematic illustration of the effect of high electric field regions on the catalyst surface in attracting LiPSs and facilitating Li^+^ dissociation from LiTFSI. b) Framework and cube structure model in finite element simulation. c,d) Simulated electric field distribution around the framework (c) and cube (d) models along the X, Y, and Z‐axes at 2.35 V. e) Simulated electric field distribution in the XY plane of the framework at 2.35 V. The arrows indicate the electric field distribution around the models, where the size and direction of each arrow represent the magnitude and direction of the electric field. f) Visualization of the charge density distribution on the models.

The Gibbs free energy profile of the SRR on the Co_3_O_4_ (111) surface was investigated using density functional theory (DFT) calculations (Figure S1, Supporting Information). The results show that, except for the spontaneous exothermic conversion of S_8_ to Li_2_S_8_ (−3.53 eV), all subsequent LiPSs conversion steps are endergonic. Specifically, the calculated Gibbs free energy changes are 0.37 eV for Li_2_S_8_ → Li_2_S_6_, 0.34 eV for Li_2_S_6_ → Li_2_S_4_, 0.58 eV for Li_2_S_4_ → Li_2_S_2_, and 0.96 eV for Li_2_S_2_ → Li_2_S. Among these, the liquid‐phase conversion of Li_2_S_8_ to Li_2_S_6_ exhibits a relatively high energy barrier, while the liquid‐to‐solid transition from Li_2_S_4_ to Li_2_S_2_ shows an even higher one. Notably, due to sluggish diffusion, the solid‐state conversion of Li_2_S_2_ to Li_2_S possesses the highest energy barrier, identifying it as the rate‐determining step (RDS) in the SRR process. These substantial energy barriers to LiPSs conversion significantly hinder the kinetics of the SRR. In the LSBs, the electric field force attracts negatively charged LiPSs to move to the high electric field region. Therefore, achieving electric field enhancement on the catalysts’ surface will facilitate the mass transfer concentration of reactants at the active sites, thereby optimizing the SRR and inhibiting the shuttling effect of LiPSs. Moreover, this electric field enhancement effect facilitates the effective concentration of TFSI^−^ anions on the catalyst surface, promotes the release of Li^+^ from LiTFSI, and thereby enhances the transport efficiency of Li^+^ within the battery (**Figure** **1**a).

To validate the hypothesis that the nano‐framework structure enables 3D electric field enhancement, finite element simulations were conducted to examine the electric field distribution in different structures under varying voltage conditions. Cube and nano‐framework models are first constructed according to the characteristics of the Co_3_O_4_‐based catalysts. The sizes are all 500 × 500 × 500 nm, and the framework edge is 100 nm (Figure 1b). The electric field intensities are simulated at 2.35 and 2.05 V respectively. First, upon applying voltage along the X, Y, and Z axes of the models, it was observed that the framework structure exhibited higher electric field intensity at the tips in all dimensions compared to the cubic model, along with a larger distribution range of high electric field intensity regions (Figure 1c,d; Figure S2a,b, Supporting Information). We additionally constructed a nano‐cone model to simulate the conventional tip‐induced electric field enhancement for comparison. As shown in the simulation, when the bias is applied along the Z direction, a strong electric field is only observed at the apex of the cone, while the remaining regions exhibit negligible enhancement. In contrast, when the voltage is applied along the X or Y direction, no significant field enhancement is generated on the cone structure, further confirming the highly directional nature of the traditional tip effect. In comparison, the nano‐framework achieves electric field amplification simultaneously along multiple planes and spatial axes due to the continuous distribution of sharp‐edge nodes throughout the 3D network (Figures S2c and S3, Supporting Information). This unique characteristic of electric field enhancement across multiple planes in 3D directions has not been reported in previously studied materials. Further analysis of the electric field distribution in the XY plane under a voltage applied along the X axis reveals a significant increase in electric field intensity at the sharp angular regions. As shown in Figure 1e and Figure S4 (Supporting Information), at 2.35 and 2.05 V, the electric field intensity around the framework is enhanced by 1.2 times compared to the cubic model. This localized electric field enhancement originates from the concentrated charge density on the material's surface. For example, when voltage is applied along the Z‐axis, the point charge density at the edges of the framework is significantly higher (Figure 1f; Figure S5, Supporting Information). This observation confirms that the multi‐angular tips of the framework enhance the concentration of charge density, resulting in a stronger electric field intensity, which is conducive to improving the mass transfer efficiency of charged species on the catalyst surface.

**Figure 2:**
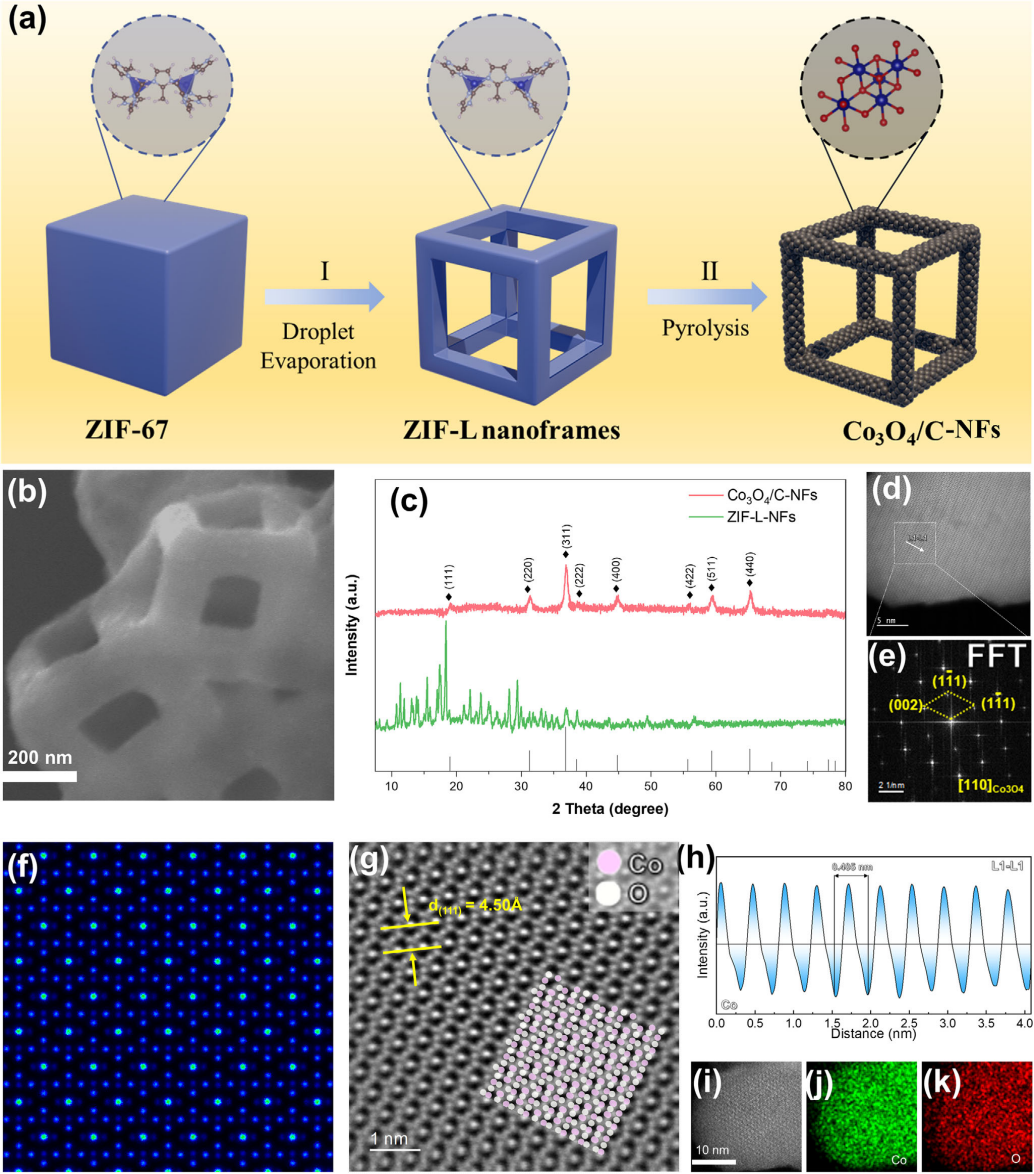
a) Schematic illustration of the synthesis of Co_3_O_4_/C‐NFs. b) SEM image of Co_3_O_4_/C‐NFs. c) XRD patterns of ZIF‐L NFs and its derivatives. d) HAADF‐STEM image through [110] and e) FFT pattern of the white rectangle selection of a Co_3_O_4_/C‐NFs nanocrystal. f) STEM simulation pattern of Co_3_O_4_ along the [110] axis. g) Enlarged HAADF‐STEM images and the atomic arrangement of the white rectangle in (e). h) corresponding line intensity profiles along the L1‐L1 selection in (e). i–k) HAADF‐STEM image and elemental mappings for a Co_3_O_4_ nanoparticle.

To prepare materials with nano‐framework structures, we first prepared ZIF‐L with nano‐framework (ZIF‐L NFs) structure using cubic ZIF‐67 as precursors according to our previous work.^[^ ^27^ ^]^ Finally, we obtained Co_3_O_4_‐based materials (Co_3_O_4_/C‐NFs) with a nano‐framework structure by pyrolyzing ZIF‐L NFs in air (**Figure** **2**a). For comparison, we also pyrolyzed ZIF‐67 to prepare cubic Co_3_O_4_‐based materials (Co_3_O_4_/C‐NC) (Figure S6, Supporting Information). Field emission scanning electron microscopy (FE‐SEM) images reveal that the average size of the ZIF‐L NFs is ≈500 nm. While FE‐SEM further confirmed that Co_3_O_4_/C‐NFs retained the unique nano‐framework structure (Figure 2b). XRD analysis characterized the crystal structure of the synthesized materials, indicating a successful transformation of the crystal structure after pyrolysis (Figure 2c). The obtained Co_3_O_4_/C‐NFs shows good agreement with the typical FCC structure of Co_3_O_4_ (PDF# 43‐1003). High‐resolution transmission electron microscopy (HR‐TEM) of Co_3_O_4_/C‐NFs showed that the diameter of Co_3_O_4_ particles is mainly uniformly distributed ≈10 nm (Figure S7a, Supporting Information). Moreover, the HR‐TEM image of Co_3_O_4_/C‐NFs (Figure S7b, Supporting Information) showed clear lattice fringes with spacing of 0.243 and 0.285 nm, which can be respectively classified as the (311) and (220) crystal planes of Co_3_O_4_. In addition, high‐angle annular dark field scanning transmission electron microscopy (HAADF‐STEM) images and elemental mapping showed that Co, C, and O are uniformly distributed and dispersed in the materials (Figure S8, Supporting Information).

In order to obtain detailed structural information of Co_3_O_4_ nanoparticles, aberration‐corrected high‐angle annular dark‐field scanning transmission electron microscopy (AC‐HAADF‐STEM) was employed. Figure 2e shows the nanoparticles of Co_3_O_4_ placed along the [110] direction, and their fast Fourier transform (FFT) results identify the structure corresponding to Co_3_O_4_ (Figure 2d). The STEM image (Figure 2f) of the Co_3_O_4_ [110] axis was simulated and found to be consistent with the experimental results. As shown in Figure 2g, the nanoparticles in this area along the [110] axis are composed of periodically activated pure Co and O, and the (111) crystal plane spacing can be observed. The line intensity distribution along the white dashed rectangular area in Figure 2h further confirms the formation of ordered Co_3_O_4_. In addition, the uniform distribution of Co and O elements can also be seen in the elemental mapping of a single Co_3_O_4_ particle (Figure 2i–k). The above proves that the framework material of the Co_3_O_4_ particle was successfully prepared.

Additionally, Raman spectroscopy analysis (Figure S9, Supporting Information) revealed distinct Raman shift peaks correspond to the F_2g_, E_g_, and A_1g_ peaks of Co‐O for both Co_3_O_4_/C‐NFs and Co_3_O_4_/C‐NC. X‐ray photoelectron spectroscopy (XPS) was employed to investigate the influence of surface chemical composition and electronic states on Co_3_O_4_/C‐NFs and Co_3_O_4_/C‐NC (Figures S10 and S11, Supporting Information). The full spectrum revealed the presence of peaks corresponding to Co, O, and C. Notably, the Co/O ratio was found to be ≈3:4 (Table S1, Supporting Information). As shown in Figure S12 (Supporting Information), the high‐resolution Co 2p XPS spectra for the two catalysts can be deconvoluted into two sets of peaks corresponding to Co^2+^ (796.2 and 780.8 eV) and Co^3+^ (794.5 and 779.2 eV).^[^ ^28^ ^]^ The positions of the Co 2p peaks for both samples are similar. The above results demonstrate that the successful preparation of Co_3_O_4_‐based catalysts possess the same structural features.

**Figure 3:**
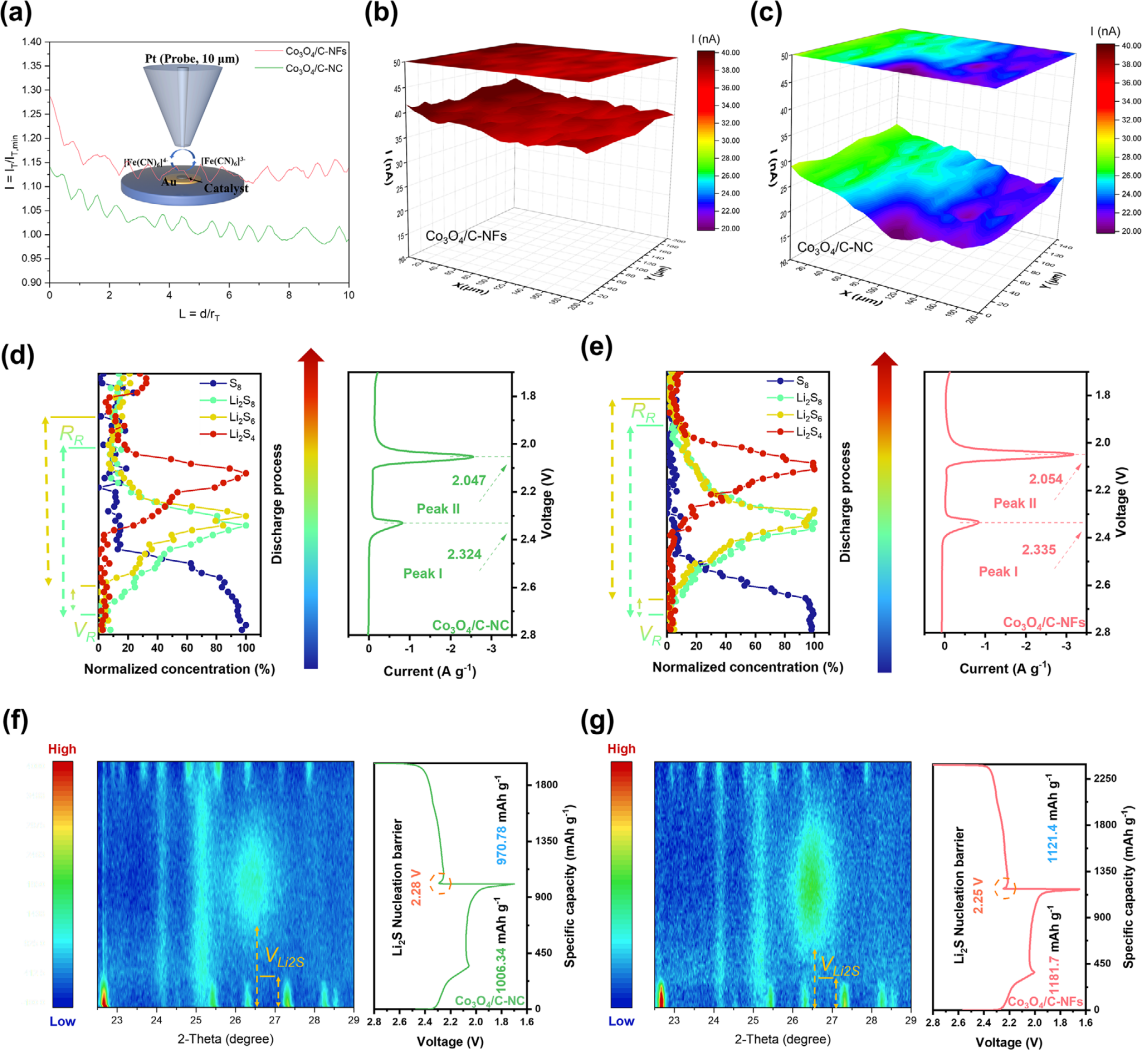
a) SECM tip approach curves (PACs) of Co_3_O_4_/C‐NC and Co_3_O_4_/C‐NFs (Inset: the charge transfer process at the interface of the UME/substrate electrode). b,c) SECM images of (b) Co_3_O_4_/C‐NFs and (c) Co_3_O_4_/C‐NC at a constant height within a geometric area. d,e) The content changes of different staged LiPSs and the corresponding CV curves of the Li‐S cells assembled by (d) Co_3_O_4_/C‐NC @PP and (e) Co_3_O_4_/C‐NFs@PP during in situ Raman tests. f,g) In situ XRD characterization in contour plots of Li‐S cells and corresponding change‐discharge profiles with (f) Co_3_O_4_/C@PP and (g) Co_3_O_4_/C‐NFs@PP during the initial cycle at 0.1C.

To further validate the presence of the 3D electric field enhancement in our material, we employed high‐resolution scanning electrochemical microscopy (SECM) to probe the field enhancement within the micro‐/nanoscale range. As illustrated in the inset of **Figure** **3**a, a Pt ultramicroelectrode (UME) probe and the catalyst electrode were immersed in an electrolyte containing a redox mediator. The UME recorded the steady‐state tip current generated by the substrate–tip redox cycling at a fixed working distance. The cyclic voltammogram (CV) of the tip (Figure S13, Supporting Information) exhibited a characteristic sigmoidal shape, confirming stable probe performance and reflecting the electrochemical reactivity of the electrode surface. As shown in Figure 3a, when the Pt tip approached the substrate vertically, the tip current gradually increased, indicating a positive feedback mode that reflects the induced current generated near the catalyst surface. Throughout the approach process, Co_3_O_4_/C‐NFs consistently displayed a higher tip current than Co_3_O_4_/C‐NC, with a more pronounced current jump near zero distance. This behavior indicates the presence of a stronger localized electric field surrounding the nano‐framework, thereby experimentally confirming the field enhancement effect predicted by our simulations before.

We further employed SECM mapping under a scan area of 1 µm × 1 µm to evaluate the material's ability to facilitate anion mass transfer and adsorption. The distance between probe and catalysts was fixed at 10 µm. During the scan, the tip was rastered across the substrate membrane while recording the current variations arising from microelectrochemical activity between the tip and the substrate surface. These current values were then converted into 3D maps (Figure 3b,c). Notably, the Co_3_O_4_/C‐NFs exhibited a pronounced increase in reaction current, indicating a stronger anion adsorption capability, whereas the Co_3_O_4_/C‐NC counterpart showed a weaker response, resulting in a lower surface current intensity. To further understand the intrinsic electrochemical and mass transfer characteristics of these catalysts, the electrochemical surface areas (ECSA) were estimated from the electrochemical double‐layer capacitance (C_dl_), determined at various CV scan rates (10, 20, 30, 40, and 50 mV s^−1^), as shown in Figure S14 (Supporting Information). The C_dl_ values of Co_3_O_4_/C‐NFs and Co_3_O_4_/C‐NC were 3.18 and 3.05 mF cm^−2^, respectively, indicating that the enhanced current response of Co_3_O_4_/C‐NFs does not originate from differences in surface area. Moreover, benefiting from the 3D electric field enhancement, the nano‐framework exhibited a more uniform current distribution over a spatial range of 200 µm × 200 µm, further highlighting its superior charge‐transfer characteristics.

In order to explore the enhancement mechanism of the framework structure on SRR kinetics, finite element analysis was employed to investigate the effect of the 3D electric field enhancement induced by the framework on the mass transfer of LiPSs at the catalyst surface. To estimate the quantitative influence of the electric field on the surface adsorption anion concentration, the Gouy–Chapman–Stern model is used to simulate the ion density of LiPSs adsorbed on the surface of the material. At 2.35 and 2.05 V, the concentration of LiPSs around the framework model is 1.1 times that of the cube (Figure S15, Supporting Information). Due to the 3D electric field enhancement, LiPSs can achieve rapid mass transfer on the catalyst surface, thus achieving higher concentrations. For the SRR, elevated concentrations of LiPSs will significantly enhance the rate of conversion. Based on this result, we speculate that in the actual SRR system, the 3D electric field enhancement effect induced by the framework structure can increase the local LiPSs concentration, thereby significantly improving the SRR conversion efficiency. To verify the above speculation, we assembled symmetric LiPSs batteries using PP with different functionalizations as separators, and systematically investigated its effects on the catalytic conversion kinetics of LiPSs. At 8 mV s^−1^, the symmetrical cells assembled with Co_3_O_4_/C‐NFs@PP exhibited smaller polarization and higher redox current (Figure S16, Supporting Information), indicating a higher degree of internal electrochemical reactions, suggestive of a significant enhancement in the kinetic conversion process of LiPSs. To further verify the promotion effect of different catalysts on SRR, Co_3_O_4_/C‐NC and Co_3_O_4_/C‐NFs electrodes were constructed for Li_2_S deposition tests to compare their effects on the catalytic conversion of liquid‐phase LiPSs to solid‐phase Li_2_S (Figure S17, Supporting Information). It was observed that on the Co_3_O_4_/C‐NFs electrode, Li_2_S nucleation exhibited the highest current peak and the fastest deposition rate, indicating a considerable improvement in the liquid–solid phase conversion ability of SRR. Additionally, the amount of Li_2_S deposited on the Co_3_O_4_/C‐NFs electrode, calculated via the Faraday process, was significantly higher than that on the Co_3_O_4_/C‐NC and black electrode. These results confirmed that Co_3_O_4_/C‐NFs with its unique framework structure can increase the mass transfer efficiency, activate long‐chain LiPSs, and thus accelerate electron transfer kinetics during the SRR.

To clearly and conveniently describe the impact of the frame structure on mass transfer and transformation of LiPSs during the SRR, we developed a universal method for evaluating the kinetics of SRR steps, based on in situ characterization. First, in situ Raman spectroscopy and synchronous CV tests (Figure 3d,e; Figures S18 and S19, Supporting Information) were employed for real‐time species characterization and electrochemical peak matching to evaluate the kinetics of the conversion from Li_2_S_8_ to Li_2_S_6_. Based on the signals of LiPSs observed in the in situ Raman tests, the onset time of the Li_2_S_x_ signals is defined as *T_g_*. When the ratio value of *T_g(Li2S6)_*/T*_g(Li2S8)_* approaches 100%, it implies that the production rates of Li_2_S_8_ and Li_2_S_6_ are more synchronous. This enhanced synchrony reflects more efficient mass transfer and faster kinetics in the conversion of Li_2_S_8_ to Li_2_S_6_. Notably, the values of *T_g(Li2S6)_*/T*_g(Li2S8)_* in cells assembled with Co_3_O_4_/C‐NFs@PP were 95.8% (Figure 3d), closer to 100% compared to those with Co_3_O_4_/C‐NC @PP (89.6%) and PP separators (85.4%) (Figure 3e; Figure S15, Supporting Information), indicating that Co_3_O_4_ with unique framework structure can increase the mass transfer efficiency and effectively enhance the conversion kinetics of Li_2_S_8_ to Li_2_S_6_. Furthermore, the duration of the Li_2_S_x_ signals is defined as *E_R_*, provides additional insight into polysulfide behavior. The ratio values of *E_R(Li2S6)_*/*E_R(Li2S8)_* in cells assembled with Co_3_O_4_/C‐NFs@PP are much closer to 100% compared to other cells, indicating that less Li_2_S_8_ shuttles to the negative side, which has a certain promotion effect on electrode protection and lithium deposition on the negative side. Additionally, the CV diagrams corresponding to the in situ Raman characterization displayed two reduction peaks at the peak times of Li_2_S_6_ and Li_2_S_4_ content. These results aligned with the characteristics of the double discharge platform in LSBs. For cells assembled with Co_3_O_4_/C‐NFs@PP, reduction peaks were observed at 2.335 and 2.054 V, with the peak currents of 0.835 and 3.176 A g^−1^, respectively, which occurred earlier and were higher than those in other cells. These peak potentials and peak current values confirmed the enhanced SRR kinetics in the Co_3_O_4_/C‐NFs@PP assembled cells.

Further, in situ XRD characterization was employed to characterize the cells and evaluate the kinetic process of the formation of Li_2_S (RDS). The disappearance time of S_8_ (*D_S8_*) and the onset time of Li_2_S (*T_g(Li2S)_*) were detected. The ratio of these times effectively reflects the conversion rate of Li_2_S in the reduction reaction (Figure 3f,g; Figure S20, Supporting Information). It was observed that the *D_S8_/T_g(Li2S)_* value in cells assembled with Co_3_O_4_/C‐NFs@PP was 0.581 (Figure 3f), significantly higher than that of cells assembled with Co_3_O_4_/C‐NC @PP (0.458) and PP (0.364) (Figure 3 g; Figure S20, Supporting Information), indicating that Co_3_O_4_‐NF/C with 3D electric field enhancement features increases the mass transfer efficiency of LiPSs and thus enhances reduction kinetics, further accelerates the formation of Li_2_S from the onset of the SRR. During discharge, characteristic peaks belonging to α‐S_8_ appeared at 23.2°, 25.8°, 26.7°, 27.6°, and 28.6°. As the reaction progressed, S_8_ transformed into liquid high‐order LiPSs. After discharge entered the second plateau, a characteristic peak for solid Li_2_S emerged near 26.9°. Due to improved nucleation efficiency, the Li_2_S signal in cells assembled with Co_3_O_4_/C‐NFs@PP appeared earlier, was stronger, and the reaction was more complete. Additionally, the charge–discharge curve showed a decrease in the nucleation potential of the improved cells (2.25 V). The effective reduction of the shuttle effect resulted in the modified cells exhibiting a much higher discharge and charge specific capacity (1181.7 and 1121.4 mAh g^−1^) compared to Co_3_O_4_/C‐NC assembled cells (1006.34 and 970.78 mAh g^−1^) and PP assembled cells (905.5 and 817.2 mAh g^−1^). The in situ characterization strategy not only reveals the real process of the internal electrochemical reaction but also quantitatively and visually demonstrates the strength of the reduction kinetics through data analysis and processing. The above characterization results and numerical analysis strongly confirm that the nano‐frame structure catalyst has a significant improving effect on SRR kinetics. The in situ characterization results mentioned above strongly demonstrate that the framework‐structured Co_3_O_4_ exhibits excellent kinetics during the conversion of various LiPSs in the SRR. This is consistent with the simulation, proving that the 3D electric field enhancement brought by the framework structure can promote the mass transfer of LiPSs to the catalyst surface, thereby enhancing the SRR kinetics.

**Figure 4:**
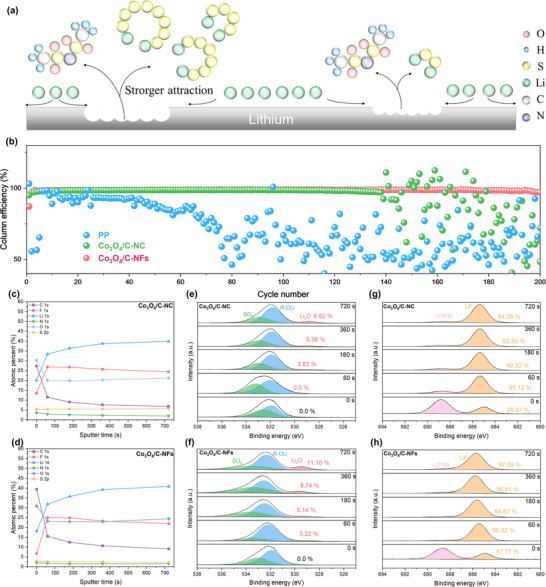
a) Schematic illustration of the morphology destruction of lithium deposition on the electrode surface due to the additional consumption of Li^+^ by LiPSs anions. b) The coulombic efficiency performance of Li‐Cu cells with Li_2_S_6_ additives in the lithium electrode side assembled with different interlayers cycling at 1 mA cm^−2^, 1 mAh cm^−2^. c,d) Depth profiles of SEI layers on Li anode surface in Li||Li cells after cycling at 1 mA cm−2, 1 mAh cm−2 for 20 cycles with Co_3_O_4_/C‐NC @PP (c) and Co_3_O_4_/C‐NF@PP (d); O 1s e,f) and F 1s g,h) XPS spectra of SEIs from Li‖Li symmetrical cells with different catalysts after cycling tests.

Furthermore, a comprehensive investigation was conducted to explore the impact of the 3D electric field enhancement, which is induced by the unique framework structure, on Li^+^ mass transfer. On one hand, the transport of Li^+^ is significantly influenced by the shuttle effect of LiPSs, and on the other hand, it is notably constrained by the dissociation efficiency of LiTFSI (**Figure** **4**a). First, a series of Li‐Cu cells were assembled to investigate the effects of diverse LiPSs on Li deposition behavior (Figure S21, Supporting Information). The presence of Li_2_S_8_ disrupted the original uniform deposition of Li^+^, leading to the rapid growth of Li dendrites and then immediate short‐circuiting during the deposition process. As for the cells containing Li_2_S_6_ and Li_2_S_4_, the deposition platform featured two stages, which can be attributed to the electron deposition of Li^+^ and lithiation of LiPSs. These findings suggest that high‐order LiPSs generally exhibit a stronger tendency to capture Li^+^. Such behavior potentially gives rise to causing local concentration non‐uniformities, which not only impede the normal transport of Li^+^ but also accelerate the nucleation and growth of lithium dendrites. In the preceding discussion, it was demonstrated that the electric field enhancement provided by the framework structure induces the efficient enrichment of LiPSs on the catalyst surface, thereby facilitating a rapid SRR. This effectively mitigates the shuttle effect caused by high‐order LiPSs, consequently alleviating their adverse impact on Li^+^ loss. With respect to the impact of the framework structure on the dissociation of Li^+^ from Li‐TFSI, the aggregation behavior of TFSI^−^ anions on the material surface was simulated for different structures (Figure S22, Supporting Information). The results reveal that at 2.35 V, the maximum concentration of TFSI^−^ anions around the framework structure is 1.11 times higher than that of the cube structure, with a similar ratio observed at 2.05 V. Moreover, the framework structure exhibits a more extensive distribution of high‐concentration regions on its surface. This phenomenon may be attributed to the fact that the TFSI^−^ anions easily migrate and concentrate on the catalyst surface under the influence of a strong electric field, thereby promoting the dissociation of Li^+^ from LiTFSI. As a result, the framework structure not only suppresses the shuttle effect but also significantly enhances the dissociation efficiency of Li^+^, leading to an increased Li^+^ concentration and facilitating its rapid transport to the positive electrode. To validate this conclusion, Li‖Li symmetric batteries were assembled with Co_3_O_4_/C‐NFs@PP and Co_3_O_4_/C@PP, and the transport number of Li^+^ was calculated according to the following formula (Figure S23, Supporting Information):

(2) $$t_{Li^{+}}=\frac{I_{s}\left(\Delta V-I_{o}R_{o}\right)}{I_{o}\left(\Delta V-I_{s}R_{s}\right)}$$

The migration number of the framework structure was 0.699, much higher than that of the cubic structure (0.574), which strongly demonstrates that the framework structure effectively anchors TFSI^−^ through 3D electric field enhancement, thereby significantly improving the transport capability of Li^+^.

Specific to this work, to further verify the effect of Co_3_O_4_/C‐NFs@PP on effectively inhibiting the shuttle effect of LiPSs and promoting the transport of Li^+^, thereby protecting the Li anode, a cycling test was conducted on Li‐Cu cells with LiPSs (Li_2_S_6_ as a representative) additive added on the Li electrode side. Compared to Li‐Cu cells assembled with PP and Co_3_O_4_/C‐NC@PP, it is extremely obvious that the Li‐Cu cell with Co_3_O_4_/C‐NFs@PP can stably cycle for over 200 cycles at a current density of 1 mA cm^−2^, 1 mAh cm^−2^ and maintain a coulombic efficiency close to 99%, which should be attributed to the high catalytic activity and effective physical barrier ability of nano‐framework structured Co_3_O_4_, blocking the diffusion of LiPSs to the Cu electrode side, and thus avoiding the occurrence of side reactions. (Figure 4b). To further investigate the influence of the framework‐structured materials on Li deposition behavior, we conducted systematic Li‐Cu cell deposition tests (Figure S24a–c, Supporting Information). It can be clearly seen that there are large amount of fresh and bright active lithium on the surface of the Cu electrode, indicating that the transport of Li^+^ is effectively promoted (Figure S24d, Supporting Information), and thus achieving smooth Li deposition. In contrast, the surfaces of the Cu electrode in PP and Co_3_O_4_/C‐NC@PP assembled cells are fully covered by black “dead lithium” after cycling (Figure S24e,f, Supporting Information). Further microscopic characterization of the Cu electrode's deposition morphology post‐cycling was performed by applying FE‐SEM. For the cell with Co_3_O_4_/C‐NFs@PP, it was revealed that the extremely smooth, dense, and large flakes of Li are uniformly deposited on the Cu electrode, strongly confirming a successful deposition process (Figure S24g, Supporting Information). In sharp contrast, there are crushed 5 µm thick dendritic dead Li on the Cu electrode in the Li‐Cu cell assembled with PP (Figure S24i, Supporting Information). Although the Li deposition process has been improved for the cell with Co_3_O_4_/C‐NC @PP, there are still some dead Li on the Cu electrode (Figure S24h, Supporting Information). Additionally, the Li‐S cells without LiNO_3_ were assembled to further verify the inhibitory effect of framework structure on the LiPSs shuttling (Figure S25, Supporting Information). Generally, the concentration of LiPSs reaches its maximum at a potential of 2.38 V. Consequently, the current change at a constant voltage of 2.38 V can effectively indicate the severity of the shuttle effect in cells. It was observed that the shuttle current of the cell assembled with Co_3_O_4_/C‐NFs was only 0.023 mA, suggesting that the external current required to maintain a constant voltage was the smallest, implying the weakest shuttle effect. These findings strongly demonstrated that Co_3_O_4_/C‐NFs@PP effectively promotes the transport of Li^+^ to the cathode, decelerated cells’ capacity decay, and protected the deposition electrode, achieving a safe and stable LSBs.

Electrolyte decomposition interface analysis is crucial for elucidating how changes in Li⁺ solvation structure ultimately influence deposition behavior. Post‐cycling XPS characterization revealed that the SEI formed on Co_3_O_4_/C‐NC@PP was primarily composed of LiF, whereas that on Co_3_O_4_/C‐NFs@PP exhibited a markedly higher O content (Figure 4c,d). With increasing sputtering depth, the oxygen content in the Co_3_O_4_/C‐NFs@PP SEI remained stably above 30%. Deconvolution of the O 1s spectra showed that the oxygen content reached 30.7% after 720 s sputtering, which is significantly higher than that in the Co_3_O_4_/C‐NC @PP counterpart (Figure 4e,f). Moreover, F 1s spectral deconvolution confirmed a more complete decomposition of LiTFSI (Figure 4g,h). These findings indicate that the 3D electric field enhancement in Co_3_O_4_/C‐NFs@PP reduces solvent hindrance to Li⁺ migration, facilitates anion coordination, and induces the formation of a Li‐rich SEI with superior mechanical strength and stability. Such an SEI promotes more uniform and compact Li deposition, effectively suppressing dendrite growth.

**Figure 5:**
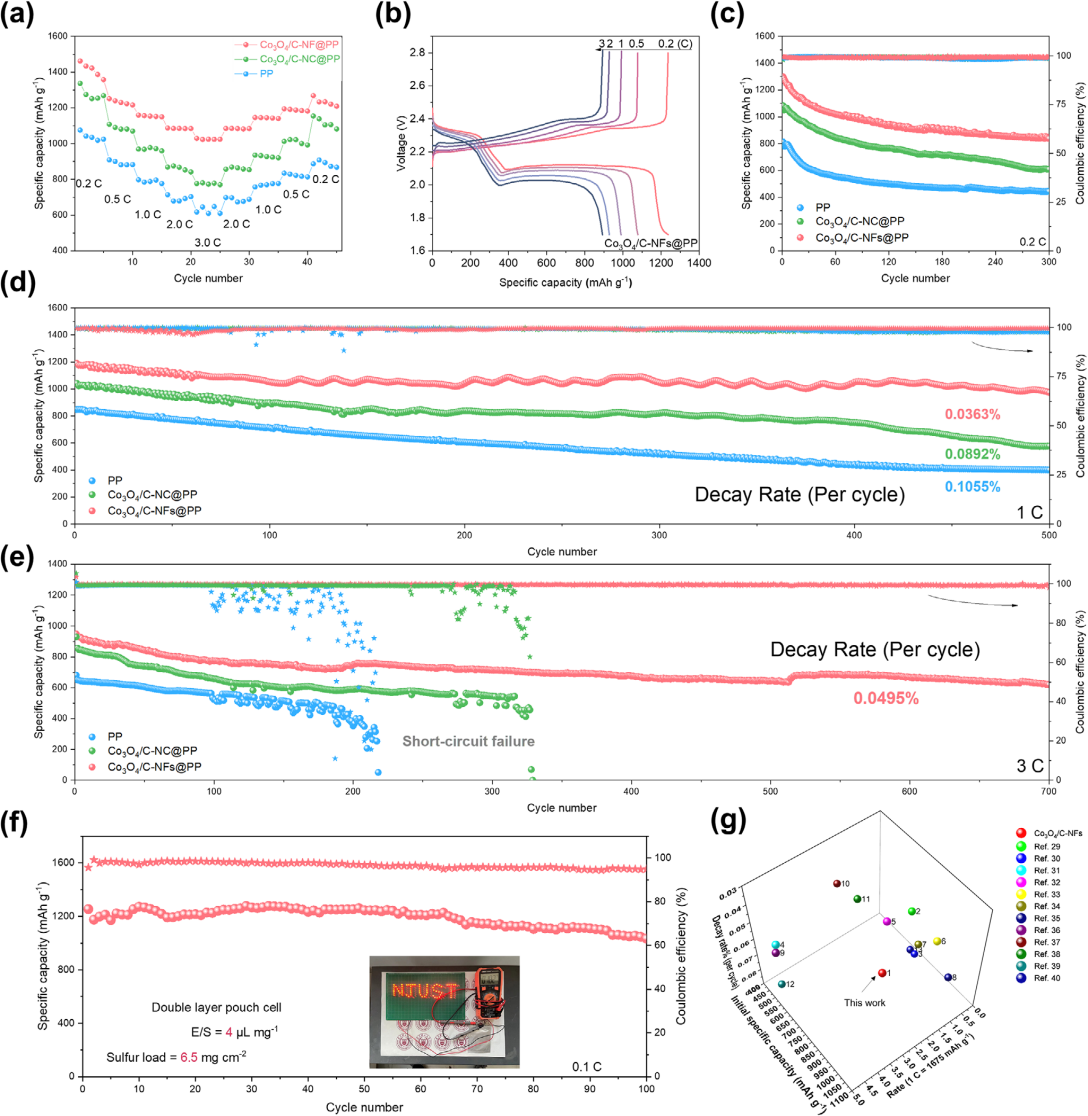
a) Rate performance of Li‐S cells assembled with different separators. b) Galvanostatic charge–discharge profiles of the Li‐S cells assembled with Co_3_O_4_/C‐NFs@PP under different C‐rates. c–e) The cycle performance of Li‐S cells assembled with different interlayers at (c) 0.2C, (d) 1C, and (e) 3C current density. f) Cycle performance of the pouch cells with Co_3_O_4_/C‐NFs@PP at 0.1C. inset, the digital photos of LED lights powered by the pouch cells with Co_3_O_4_/C‐NFs@PP. g) Performance comparison of this work with other reported improved LSBs.^[^ ^29^, ^30^, ^31^, ^32^, ^33^, ^34^, ^35^, ^36^, ^37^, ^38^, ^39^, ^40^ ^]^

To verify the stability of cells during the redox process, CV tests were first conducted at various scan rates (Figure S26, Supporting Information). It was observed that at 0.1 mV s^−1^, the reduction peaks of the cell assembled with Co_3_O_4_/C‐NFs@PP appeared earlier and the peak currents were much higher than those of other cells. Notably, as the scan rate increased, the cells with Co_3_O_4_/C‐NFs@PP exhibited the strongest anti‐polarization capability. Even at a high scan rate of 1.0 mV s^−1^, the reduction peak potential only slightly shifted, indicating high redox kinetics and rapid completion of the redox reaction within the cell. Furthermore, the rate performance of different cells was also tested to reveal the polarization phenomena during the charge and discharge process. The platform in the charge–discharge curves of the cells with PP and Co_3_O_4_/C@PP gradually diminished as the current increased from 0.2C to 3C, indicating insufficient redox kinetics to sustain normal electrochemical reactions at high currents (**Figure** **5**a). However, the cell with Co_3_O_4_/C‐NFs@PP maintained natural double platform charge–discharge curves even at 3 C, confirming their superior redox kinetics and excellent anti‐polarization properties (Figure 5b). Additionally, due to the high catalytic activity of Co_3_O_4_/C‐NFs and its effective inhibition for the LiPSs shuttle effect, the assembled Li‐S cell delivers high actual specific capacity and outstanding rate performance.

The safety and stability of cells can be reliably assessed through sufficiently long cycle tests. These tests, conducted on cells with different modified PP separators at various currents, demonstrated the stability of their internal reactions. As shown in Figure 5c–e, it was observed that Co_3_O_4_/C‐NFs@PP‐assembled cell exhibited higher specific capacity and capacity retention rates during cycle tests under varying currents. When cycling at 1C, the cycle attenuation rate of Co_3_O_4_/C‐NFs@PP‐assembled cell was only 0.0363% after 500 cycles, significantly lower than those of other cells (0.0892% for Co_3_O_4_/C@PP and 0.1055% for PP), demonstrating excellent cycle stability. Even at a higher current (3C), the cell assembled with Co_3_O_4_/C‐NFs@PP showed minimal capacity decay of 0.0495% with a retention rate of 67% after 700 cycles. In contrast, the cells with Co_3_O_4_/C@PP and PP not only experienced rapid capacity decay but also faced short‐circuit overcharge due to dendrite puncture after 200 (PP) and 330 (Co_3_O_4_/C@PP) cycles, respectively. This highlights the importance of Co_3_O_4_‐NFs@PP in effectively reducing capacity decay, and protecting the lithium anode by inhibiting shuttle effects and promoting LiPSs conversion. To verify reliability for practical applications, double‐layer pouch Li‐S cells (2 cathode and 2 anode) were assembled (Figure 5f). Under challenging conditions of 6.5 mg cm^−2^ sulfur loading and a 4 µL mg^−1^ electrolyte/sulfur ratio (E/S), the pouch cell maintained stable cycling at 0.1 C for over 100 cycles with a high specific capacity of 1200 mAh g^−1^, demonstrating promising practical application potential. Compared to previously reported catalysts,^[^ ^29^, ^30^, ^31^, ^32^, ^33^, ^34^, ^35^, ^36^, ^37^, ^38^, ^39^, ^40^ ^]^ this catalyst exhibits superior overall performance across multiple key evaluation metrics (Figure 5g).

## 3 Conclusion

Here, we have developed a 3D electric field enhancement strategy that effectively regulates the mass transfer behavior of Li^+^ and LiPSs in LSBs, resulting in improved performance. The Co_3_O_4_/C‐NFs catalyst, with its unique framework structure, utilizes 3D electric field enhancement to significantly promote the mass transfer of LiPSs toward the surface of the catalysts, thereby enhancing the SRR kinetics and inhibiting the mass transfer of LiPSs between the two electrodes, while simultaneously facilitating Li^+^ transport toward the cathode. The above experimental results and theoretical simulations strongly support these conclusions, with the developed universal evaluation method providing particularly compelling evidence for the accelerated SRR kinetics. This multiscale mass transfer regulation mechanism not only significantly enhances the battery's energy efficiency and cycling stability, but also improves its safety performance. This study provides new theoretical guidance and technical pathways for the development of LSBs based on microstructural material design.

## Conflict of Interest

The authors declare no conflict of interest.

## Supporting information



Supporting Information

## Data Availability

The data that support the findings of this study are available from the corresponding author upon reasonable request.
