# Supplementary material for: Co3O4/C‐NFs Induced 3D Electric Field Enhancement for Dual‐Regulation of Polysulfides and Li+ Transport in Lithium–Sulfur Batteries
Source: Adv Sci (Weinh). 2025 Nov 25;13(8):e20167. doi: 10.1002/advs.202520167 (PMC12884761; doi:10.1002/advs.202520167)
Supplement: Supplementary file 1 — Supporting Information [file ADVS-13-e20167-s001.docx]

**Supporting Information for**

**Co_3_O_4_/C-NFs Induced 3D Electric Field Enhancement for Dual-Regulation of Polysulfides and Li^+^ Transport in Lithium-Sulfur Batteries**

Zhijie Qi^#,a^, Jun Jiang^#,a^, Pengcheng Yao^#,^*^,b^,Zhenjie Lu^a^, Ying Shen^a^, Shujun Liu^a^, Jingwen Sun^a^, Pan Xiong^a^, Xin Wang^a^, Xiaoping Ouyang^c^, Junwu Zhu*^,a^ and Yongsheng Fu*^,a^

^a^ Key Laboratory for Soft Chemistry and Functional Materials, School of Chemistry and Chemical Engineering, Nanjing University of Science and Technology, Nanjing 210094, China

^b^ Department of Chemical and Biomolecular Engineering and Ralph O'Connor Sustainable Energy Institute, Johns Hopkins University, Baltimore, Maryland 21218, United States

^c^ Key Laboratory of Low Dimensional Materials and Application Technology, School of Materials Science and Engineering, Xiangtan University, Xiangtan 411105, China

^#^ These authors contributed equally to this work

* Corresponding Author: [pyao4@jh.edu](mailto:pyao4@jh.edu); zhujw@njust.edu.cn; fuyongsheng@njust.edu.cn

**KEYWORDS:** Nano-Frameworks, Sulfur reduction reaction, Co_3_O_4_ nano-catalysts, In-situ electrochemical technique

**Method**

*Chemicals*

All chemicals are purchased and used commercially and require no further purification.

*Synthetic procedure*

*Synthesis of ZIF-67.* Following the previous report, 0.30 g of Co(NO_3_)_2_·6H_2_O and 5 mg of CTAB were added to 10 mL of deionized water, and the mixture was sonicated for 5 minutes to form a clear solution A. Then, 4.54g of dimethylimidazole was added to 70 mL of deionized water and stirred to form solution B. Solution A was poured into solution B and stirred for 20 minutes and then to precipitate at room temperature for 24 hours. The resulting solution was centrifuged, and the obtained ZIF-67 cubes were subjected to multiple alcohol washes.

*Preparation of ZIF-L NFs.* Similar to the above steps for the synthesis of ZIF-67, but after standing for 24h, 70 mL of supernatant was removed from the solution and 10 μL of remaining mother liquor was transferred to the silicon plate (10 mm × 10 mm) at room temperature to form droplets. ZIF-L NFs were obtained after the droplets were naturally dried.

*Preparation of Co_3_O_4_/C-NFs and Co_3_O_4_/C.* The obtained ZIF-L NFs are placed in a ceramic boat, heated to 430 ℃ at a ramp rate of 2 ℃ min^-1^, and maintained for 2 h in a tube furnace under nitrogen atmosphere. After that, the furnace is cooled down to room temperature naturally to get Co_3_O_4_/C-NFs. The preparation method of Co_3_O_4_/C is similar to the above procedure, except that the precursors of pyrolysis are ZIF-67.

*Materials Characterizations*

Field emission scanning electron microscopy (FESEM, Quanta 400 FEG) equipped with energy-dispersive X-ray spectroscopy (EDS), transmission electron microscopy (TEM, JEOL JEM-2100) operated at 200 kV), and high-resolution TEM (HRTEM) (FEI Talos F200X G2) equipped with EDS (FEI SuperX G2) were used to study the microscopic morphology and crystal structure of these samples. The structure of these samples was recorded using an X-ray diffractometer Bruker D8 Advance diffractometer (Miniflex-600W, Rigaku Corporation, Tokyo, Japan) with Cu Kα (λ = 0.15406 nm) radiation over a scan angle (2*θ*) range of 5° to 80°. The Raman measurement was to record the Raman spectrum using a laser with a wavelength of 532 nm as a detector. The X-ray photoelectron spectroscopy (XPS) was carried out to analyze the element composition and binding energy of these samples surface by using Thermo ESCALAB 250 spectrometer with Al Kα (1486.6 eV) as excitation source.

*Electrochemical measurements*

Galvanostatic charge/discharge measurements were performed on a LAND CT2001A testing system with a voltage window of 1.7−2.8 V under various C-rates (1 C = 1675 mA g^-1^, based S). The autolab PGSTAT302N (Metrohm, Switzerland) electrochemical workstation was used to collect the potentiostatic, cyclic voltammetry (CV) and electrochemical impedance spectroscopy (EIS) spectra. The AC amplitude of EIS is 5 mV and the frequency ranges from 100 kHz to 0.1 Hz.

**The synthesis of cathode and assembly of battery**

The common commercial carbon nanotubes were used as cathode host materials. The active material was consisted of 80% sulfur (S) and 20% CNTs calcined in the protection of argon atmosphere at a rate of 2 ℃ min^-1^ from room temperature to 155 ℃ for 12 h. To fabricate the sulfur cathode, the as-synthesized CNT/S composites were mixed with super P and PVDF powder in a weight of 8: 1: 1 in NMP and stirred to form uniform slurry which was then casted onto an Al foil current collector and dried at 60 °C overnight. The dried cathodes were cut into disks with a diameter of 12 mm and the sulfur mass loadings were about 1.5 ~ 2 mg cm^−2^. A lithium metal disk (diameter = 15.6 mm, thickness = 450 μm) was used as the anode. The composition of electrolyte is a mixed Li-containing solution (1 M trifluoromethanesulfonimide lithium (LiTFSI) dissolved in a solution of 1, 2-dimethoxyethane (DME) and 1,3-dioxolane (DOL) (v/v = 1/1) with 1% LiNO_3_). For Li-Cu cells, different Li_2_S_n_s were added into the electrolyte as additives at a concentration of 2 mM. All CR2032 coin cells were assembled in an argon-filled glove box (< 1 ppm of O_2_ and H_2_O) and the ratios of electrolyte to S were controlled to be 15 μL mg^-1^.

**Li–Cu Coulombic Efficiency Measurement**

The Li–Cu Coulombic efficiency was evaluated in a Li|Cu half-cell configuration. During cycling, lithium was plated onto the Cu substrate at a current density of 1 mA cm⁻² with a fixed areal capacity of 1 mAh cm⁻² (corresponding to a plating time of 1 h). The subsequent stripping process was performed at the same current density until the cell voltage reached a cutoff of 1.0 V vs. Li/Li⁺. These plating/stripping steps were continuously repeated to examine the reversibility of lithium deposition and long-term Coulombic efficiency.

**The lithium ions transport rate measurements**

The Lithium ions transference number was analyzed by the combination of chronoamperometry and electrochemical impedance spectra (EIS) of Li||Li cells according to the following equation^1^:

$$t_{{Li}^{+}}= \frac{I_{s}(\Delta V-I_{0}R_{0})}{I_{0}(\Delta V-I_{s}R_{s})}$$

where *I_0_* and *I_S_* are the initial and steady-state current obtained by the chronoamperometry, respectively. *R_0_* and *R_S_* are the interfacial resistance before and after polarization measured by AC impedance analysis, respectively. *△V* is the potential difference (10 mV). The interfacial characteristic of lithium/electrolyte was investigated by the impedance analysis of Li||Li cells over storage. The frequency range for measurement was 10 mHz^-1^ MHz at ambient conditions.

**Li_2_S nucleation measurements**

For the study of liquid-solid conversion kinetics, the Ni-based ion bidirectional regulator of different qualities were dissolved in isopropanol and then dropped onto round carbon paper disks (12 mm), the total mass loading was controlled around 1 mg. 25 μL Li_2_S_8_ catholyte (0.2 M Li_2_S_8_, 1 M LiTFSI in tetreglyme) and 20 μL electrolyte without Li_2_S_8_ were added to the cathode and anode sites, respectively. The cells were first discharged to 2.06 V under a constant current of 0.112 mA and then kept potentiostatically at 2.05 V until the current dropped below 10^-5^ A. The nucleation capacity of Li_2_S can be calculated by the integral area of the plotted curve through Faraday’s Law.

**Symmetric cell assembly and kinetic evaluation of polysulfide conversion**

The symmetric cell used different carbonized samples as identical counter and working electrodes with a mass loading of 1 mg. 40 μL Li_2_S_6_ solution (0.2 M Li_2_S_6_ and 1 M LiTFSI in DOL/DME, v/v = 1/1) was used as the electrolyte. The CV measurements of the symmetric cells were performed with a voltage window between -1 to 1 V.

**Shuttle current measurements**

For the shuttle current measurement, cells with a sulfur loading of ~2 mg cm^-2^ were assembled without LiNO_3_ additive. Typically, the cells were charged and discharged 2 cycles at the current density of 0.2 C before galvanostatically charged to 2.8 V. Then the cells were discharged to 2.38 V and switched to the potentiostatic mode during which the current reached a steady-state value. This steady-state current was recorded as the shuttle current.

**In-situ optical microscope, Raman and XRD spectroscopy measurements**

The in-situ optical microscope, Raman and XRD devices were purchased from Beijing Scistar Technology Co., Ltd. Two pieces of 8 mm × 9 mm lithium sheets were used as the positive and negative electrodes of Li||Li symmetrical batteries, and the plastic with regular gap was used as the separator. The growth of dendrites on the lithium sheet during charge and discharge was observed by optical microscope at the gap.

A quartz window was used for laser passage for in-situ Raman. A hole (2 mm) was punched in the Li-metal anode to allow the laser to focus directly on the separator. The cells were run at a current density of 0.5 C. Raman signals were obtained on Renishaw Microprobe by a 532 nm laser.

As for the in-situ XRD spectrum test, a beryllium window was used for X-ray penetration; Ultrathin Al foil (~ 6 μm) was used as current collector. The sulfur loading of each cell was about 3 mg to get better signal. The cells were run at a current density of 0.1 C for in-situ XRD. Continuous scanning during cell running with each scan measured at 15-40° in 0.02° incremental steps.

*DFT calculation details*

All calculations performed within the framework of density functional theory (DFT) were implemented using the Vienna Ab initio Simulation Package (VASP).^31^ The projector-augmented wave (PAW) potential database was employed to simulate the relationship between valence electrons and atomic cores. ^32^ The electronic transfer and correlation were investigated using the Perdew-Burke-Ernzerh (PBE) exchange-correlation functional and the generalized gradient approximation (GGA). ^33, 34^ The Kohn-Sham states were expanded in a plane wave basis set with an energy cutoff of 400 eV. The convergence criteria for energy was set to 10^-5^ eV, and for forces it was set to 0.02 eV/Å during the structural optimization. The calculation employs a 2 x 2 x 1 grid mesh for K-space sampling.

For the construction of the surface model, A vacuum of 20 Å is used to eliminate the interaction between the periodic structures. According to the observation of the (111) plane of Co_3_O_4_ in the XRD pattern, the (111) plane of Co_3_O_4_ model is selected for modeling.

The Gibbs free energy profiles of reaction pathways were calculated based on the standard hydrogen electrode (SHE) model. The adsorption Gibbs free energies of reaction intermediates for each step of the SRR were obtained using the following approach.

$\Delta G= \Delta E+\Delta ZPE-T\Delta S$ (6)

Where ∆G is the binding energy of different adsorption species, ZPE is the zero point energy, T is the temperature, ∆S is the entropy change.

***Multi fields simulation details***

The distribution of electric field intensity and charged particles on the Co_3_O_4_/C-NFs and Co_3_O_4_/C catalysts were simulated by COMSOL Multiphysics. Nano-cube model and nano-frame model were established at 500 *500 *500 nm. Electric field module and dilute matter transfer module are used for simulation. The details of the simulation are as follows: (1) the applied voltages are 2.05V and 2.35V, and (2) the fluid flow particle setup has charged properties of LiPSs and TIFS^-^. (3) Transitive attributes only consider diffusion and migration

SECM Measurements

The SECM experiment was conducted using a four-electrode system controlled by a double potentiostat (Guangdong Dynechem Electronics Technology Co., Ltd., China). Pt disk electrode sealed in a glass capillary (Φ = 10 μm) is used as the SECM tip electrode, and the catalysts coated on the glass carbon (GC) disk is used as the working electrode. In order to prepare the catalysts working electrode, 2.5 mg of catalysts was dispersed in 1 mL of deionized water, ethanol, and Nafion (5 wt %) (49:49:2 by volume) and then ultrasonically treated for 30 min to form a uniform dispersion. Next, 2 μL of dispersion was dripped onto the surface of glass carbon (Φ = 3 mm) and dried naturally at room temperature. Pt wire and Hg/HgO were used as the counter electrode and reference electrode, respectively. In the experimental scheme, the feedback mode of SECM was used to execute the approach curves, keeping the potential of the SECM tip electrode at −0.3 V vs Hg/HgO and the catalysts working electrode at the open-circuit potential. The distance between the tip electrode and the catalysts working electrode is controlled at 10 μm. 0.1 M KCl was used as the supporting electrolyte in 5 mM K_3_[Fe(CN)_6_] as a mixed solution. The CV curves were recorded in the potential window of −0.1 to 0.5 V vs Hg/HgO. The CV curves and the approach curves are tested in the mixed solution. The two-dimensional scanning electrochemical microscope images of catalysts working electrode were obtained in the feedback mode. In a typical experiment, the current varied with a minute distance between the tip electrode and the surface of the catalysts working electrodes, which can be used to obtain the microscopic distribution of the electrode materials in a 3 × 3 mm^2^. Moreover, a serpentine scan was performed on the x–y plane at a 10 μm tip height. The scan step size was 10 μm, and the dwell time per pixel was 5 s.


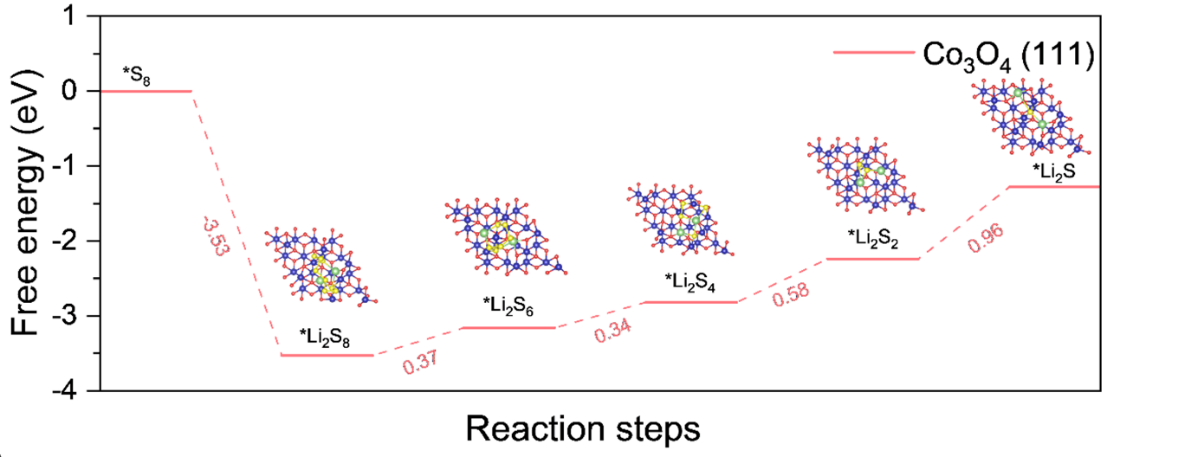


Figure S1 Gibbs free energy diagram of SRR on Co_3_O_4_.


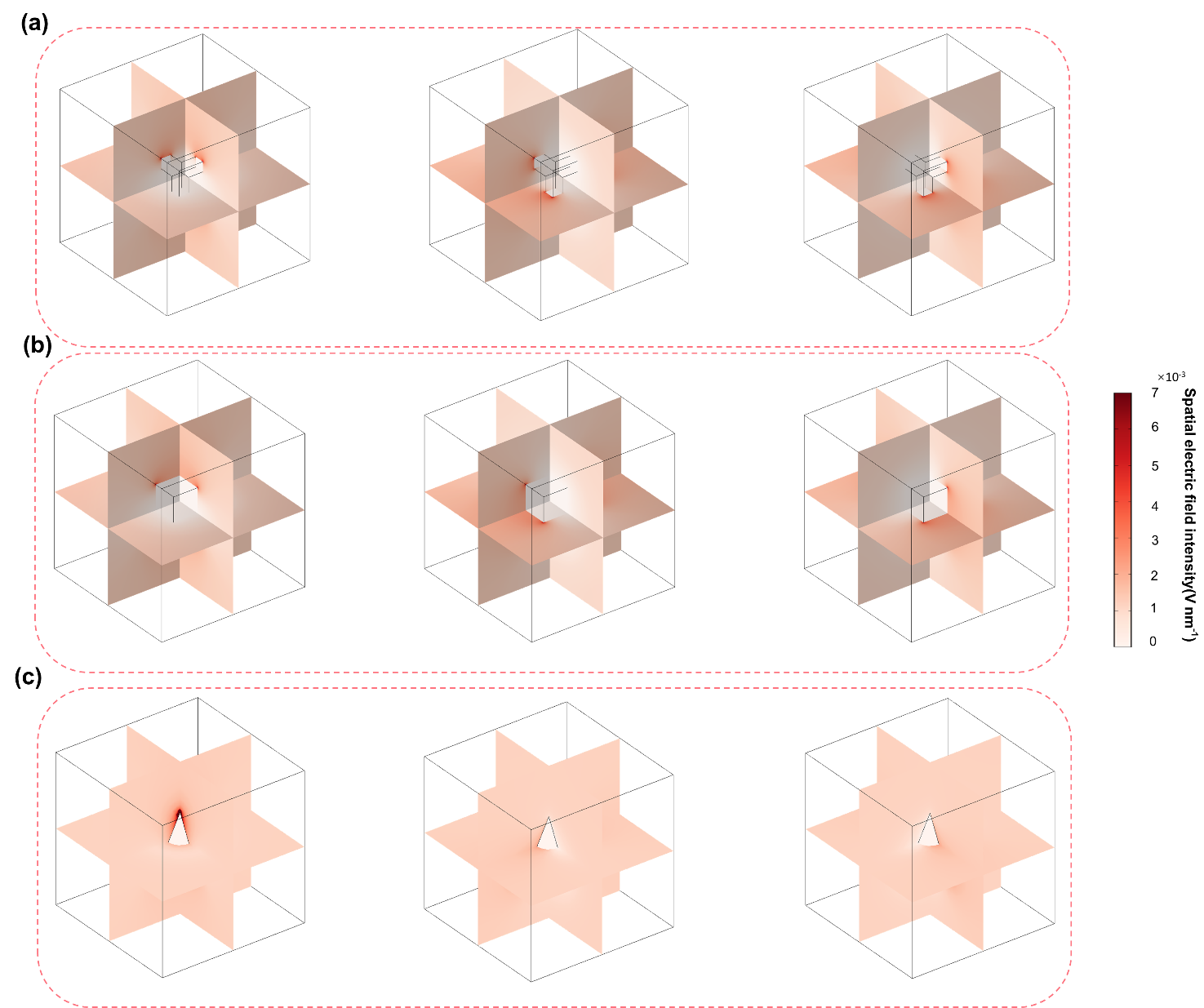


Figure S2 Simulated electric field distribution around the framework (a), cube (b), cones (c) models along the X, Y, and Z-axes at 2.05 V.


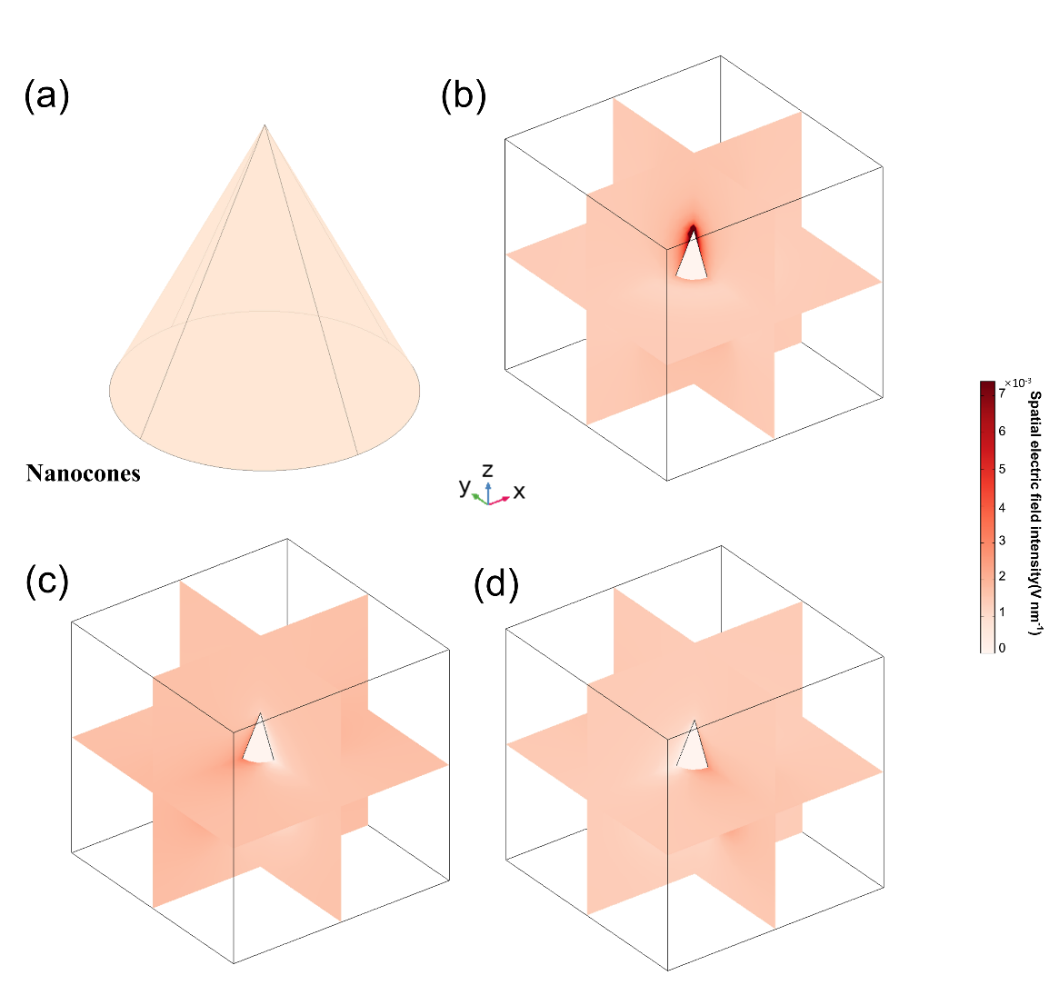


Figure S3 (a) Cones structure model in finite element simulation, (b-d) simulated electric field distribution around the cones models along the X, Y, and Z-axes at 2.35 V.


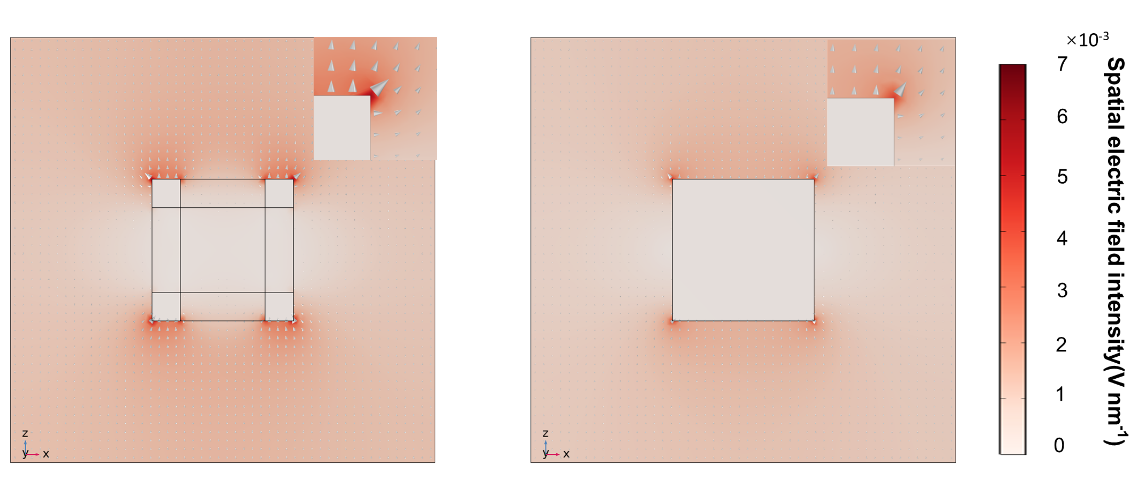


Figure S4 Simulated electric field distribution in the XY plane of the framework at 2.05 V. The arrows indicate the electrostatic field distribution around the models, where the size and direction of each arrow represent the magnitude and direction of the field.


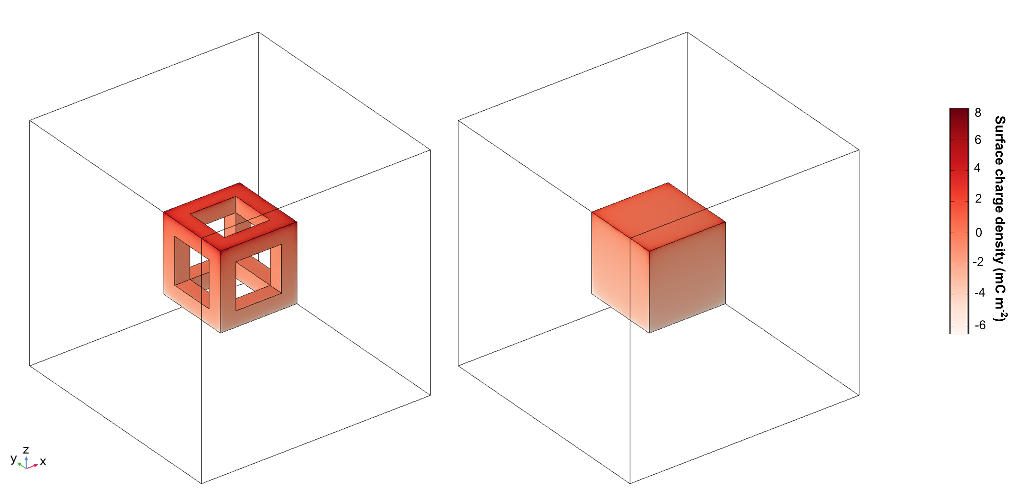


Figure S5 Visualization of the charge density distribution on the models.


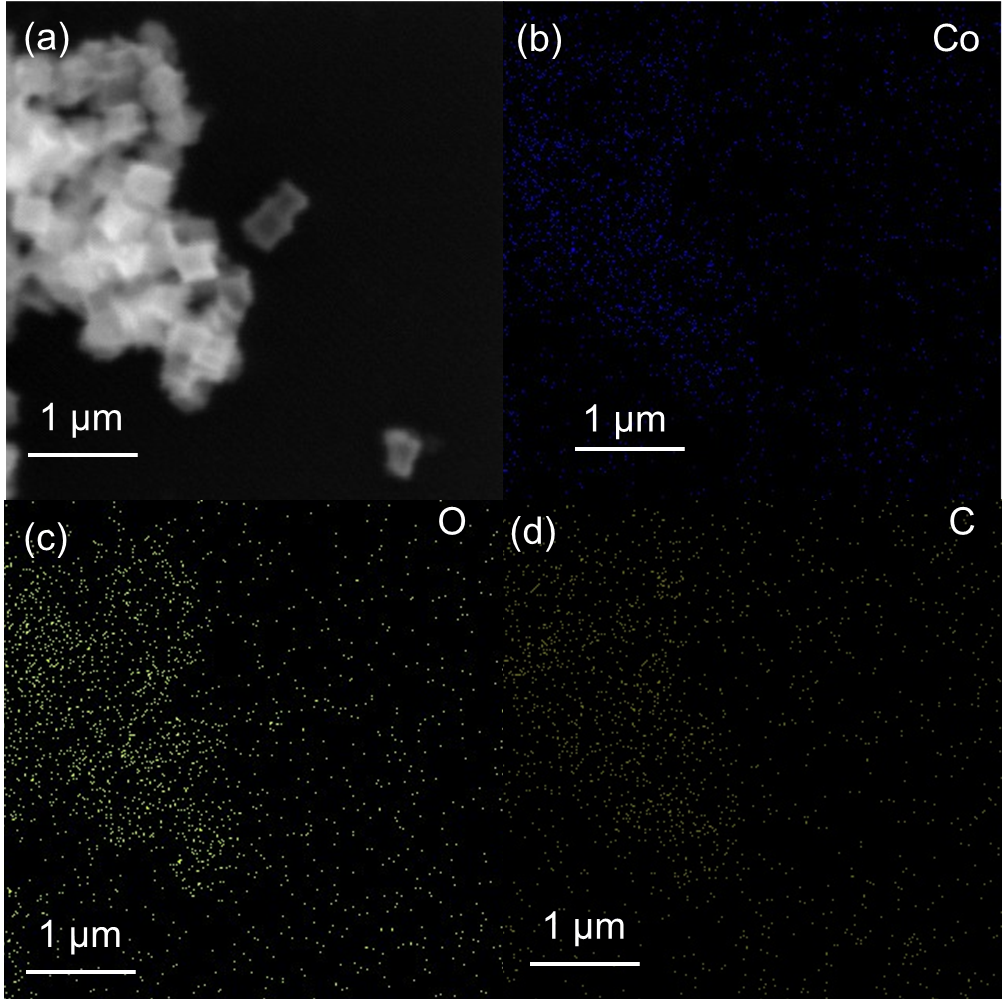


Figure S6 (a) SEM image of Co_3_O_4_/C-NC, (b-d) EDS elemental mapping of Co_3_O_4_/C-NC.


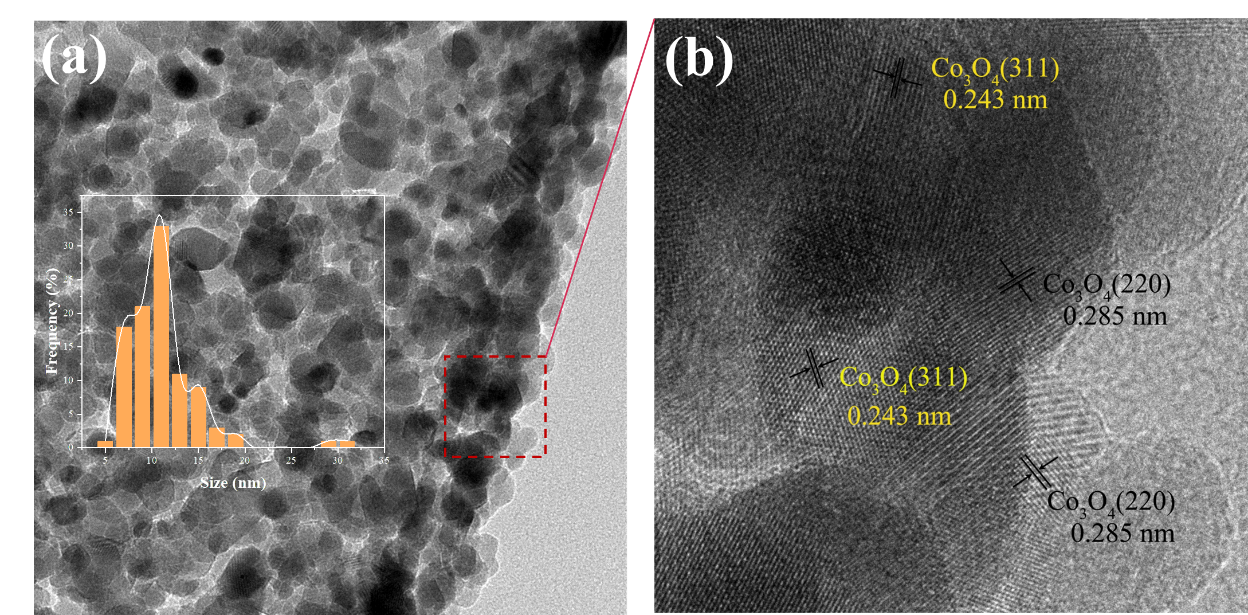


Figure S7 (a-b) HR-TEM images of Co_3_O_4_/C-NFs.


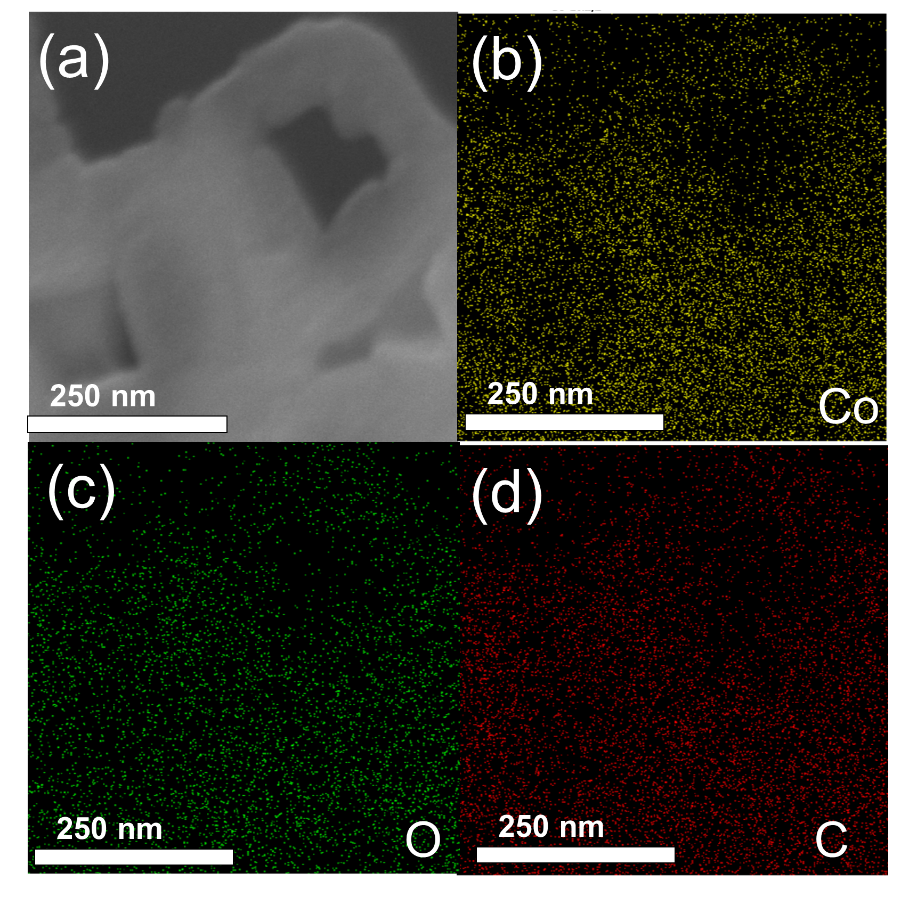


Figure S8 (a-d) EDS elemental mapping of Co_3_O_4_/C-NFs.


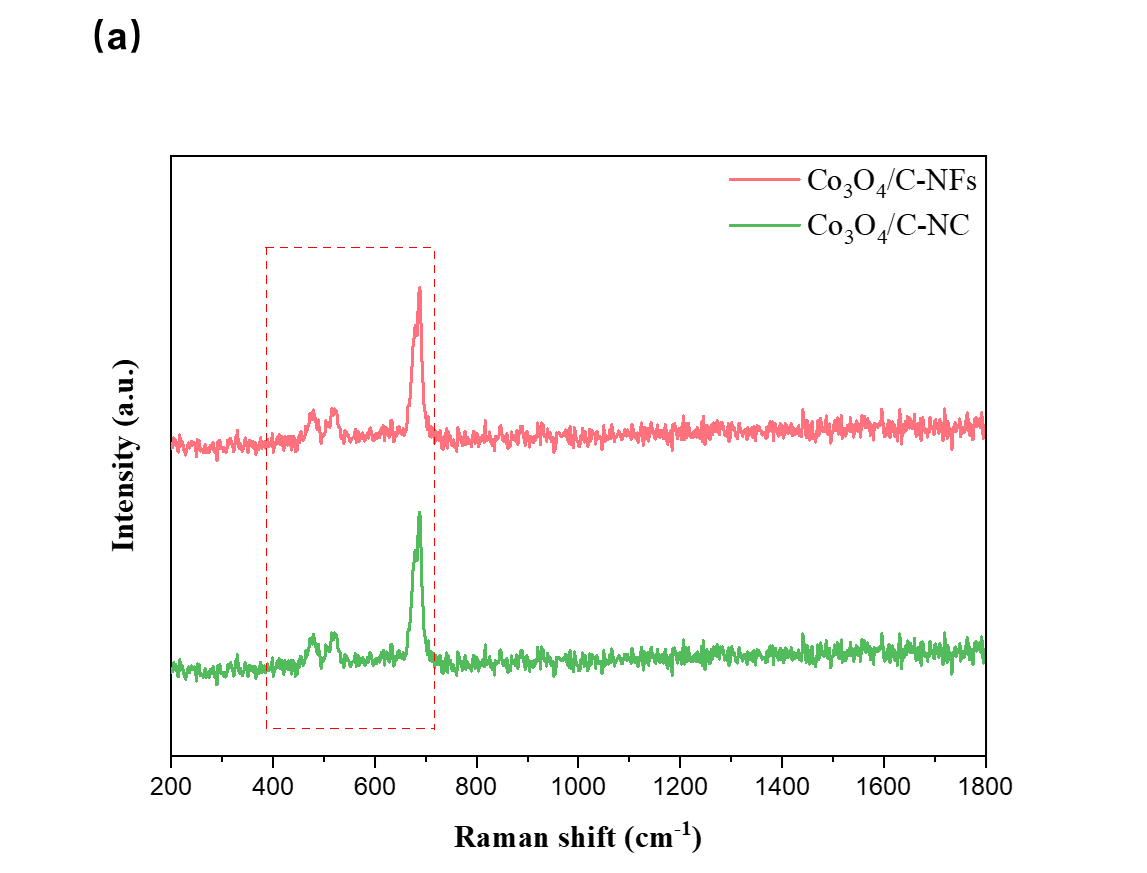


Figure S9 Raman spectra of Co_3_O_4_/C-NFs and Co_3_O_4_/C-NC.

.


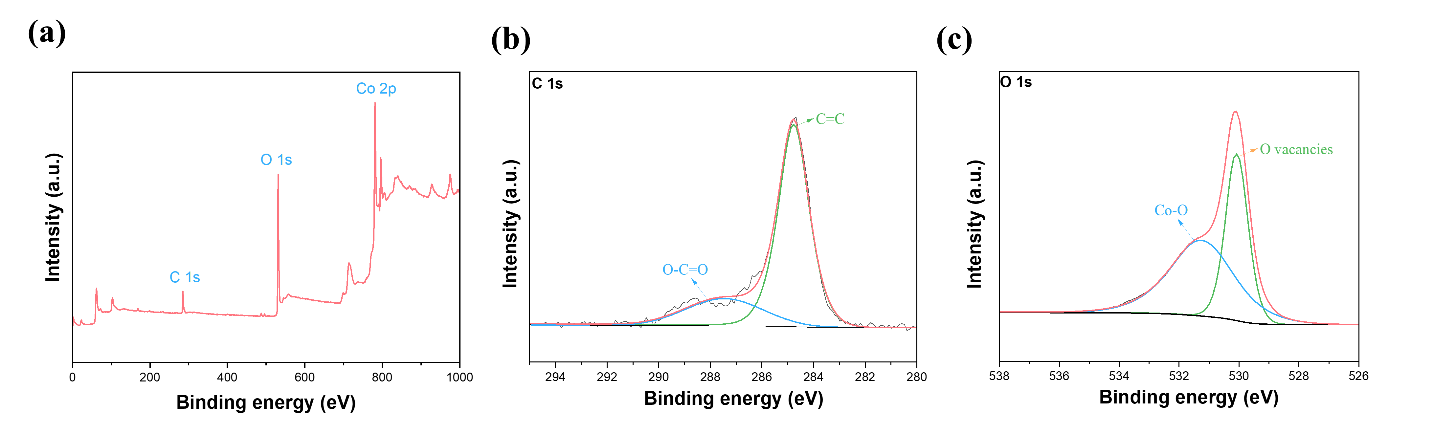


Figure S10 XPS spectroscopy of Co_3_O_4_/C-NFs.


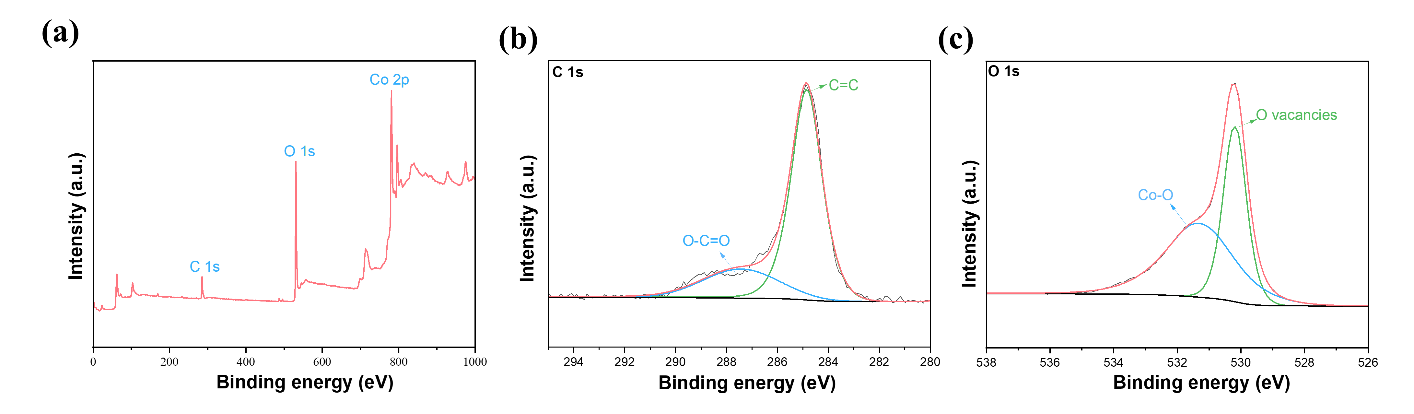


Figure S11 XPS spectroscopy of Co_3_O_4_/C-NC.


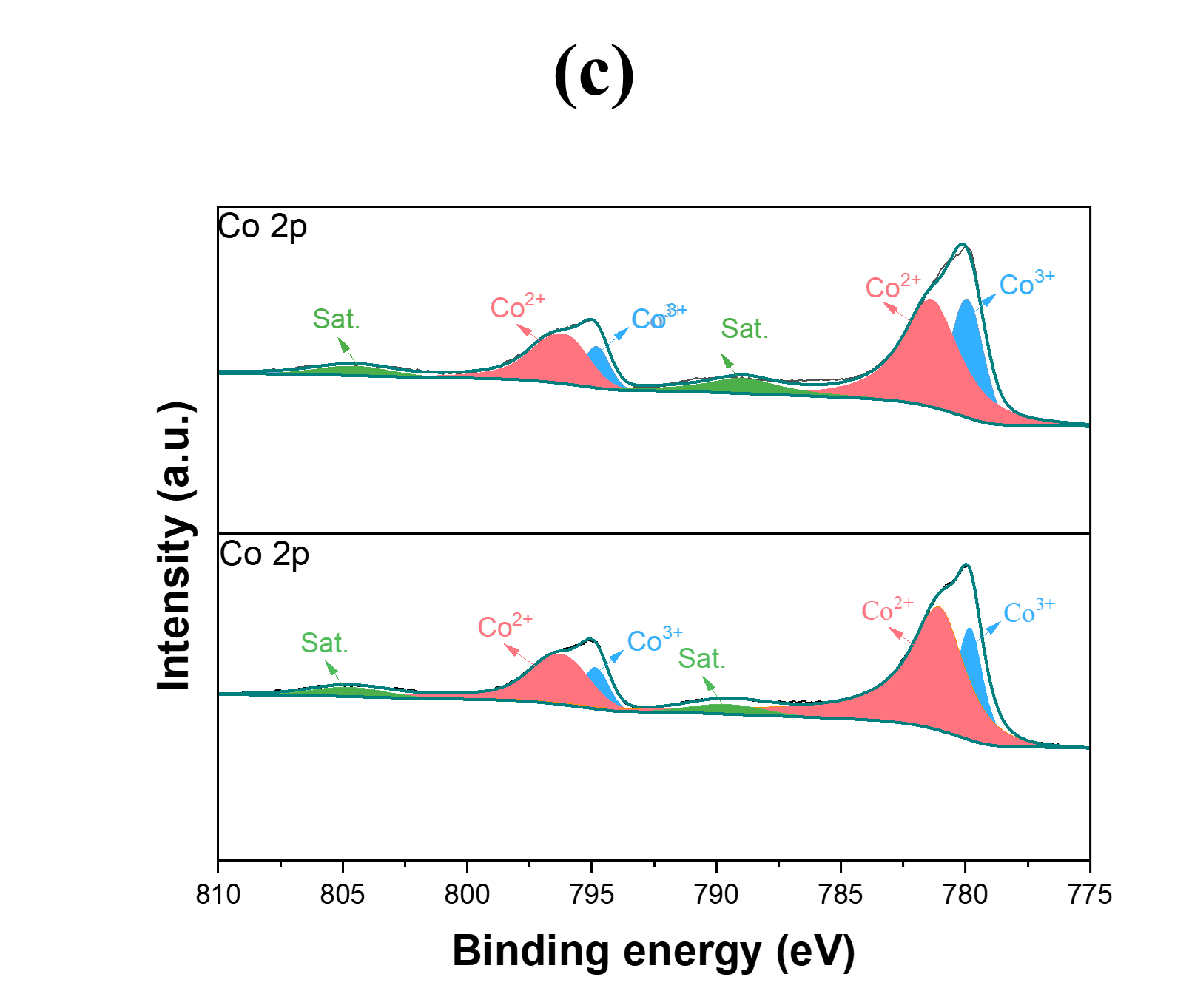


Figure S12 XPS spectroscopy of Co 2p in Co_3_O_4_/C-NFs and Co_3_O_4_/C-NC.


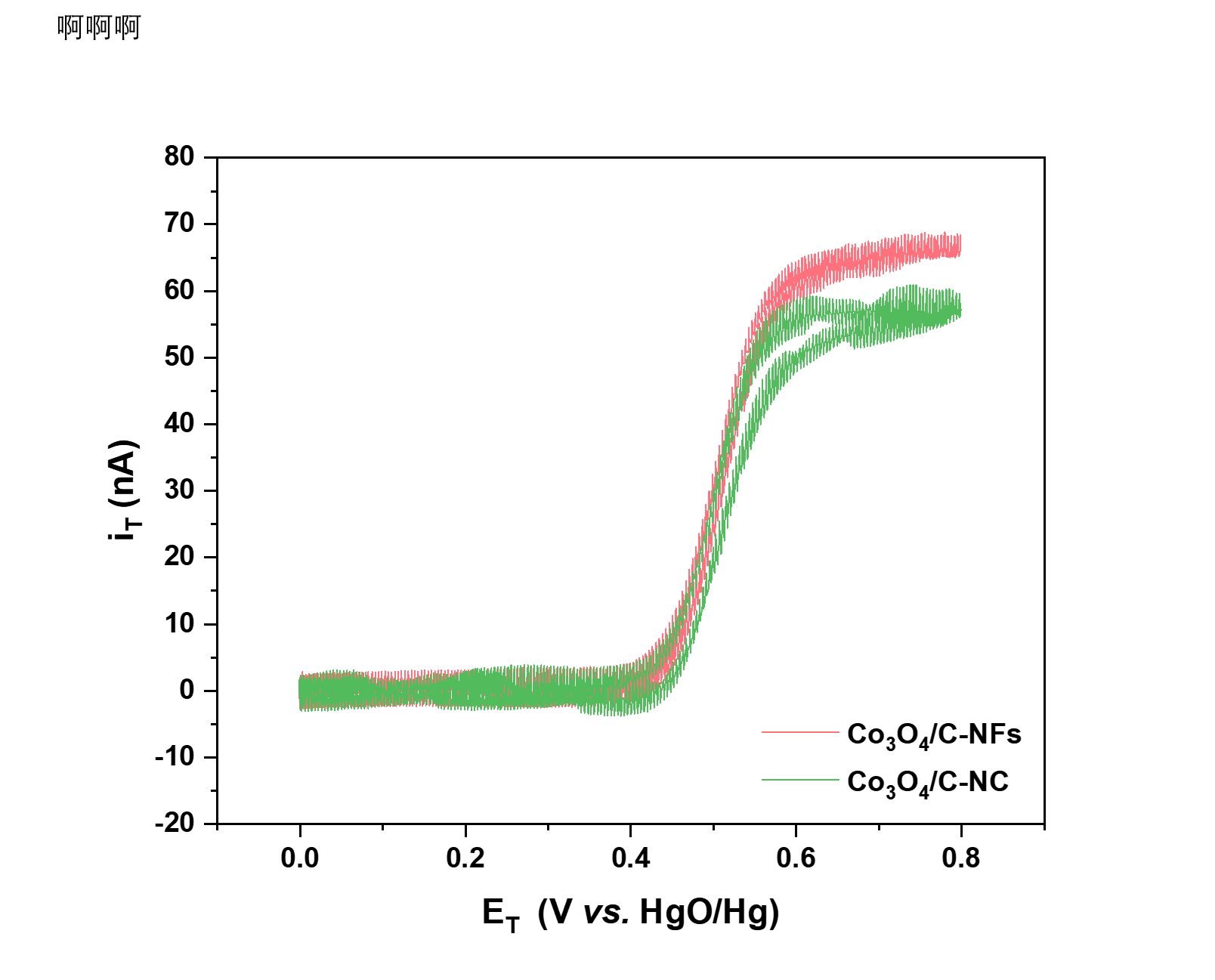


Figure S13 CVs recorded at the SECM tip over both the Co_3_O_4_/C-NFs and Co_3_O_4_/C-NC.


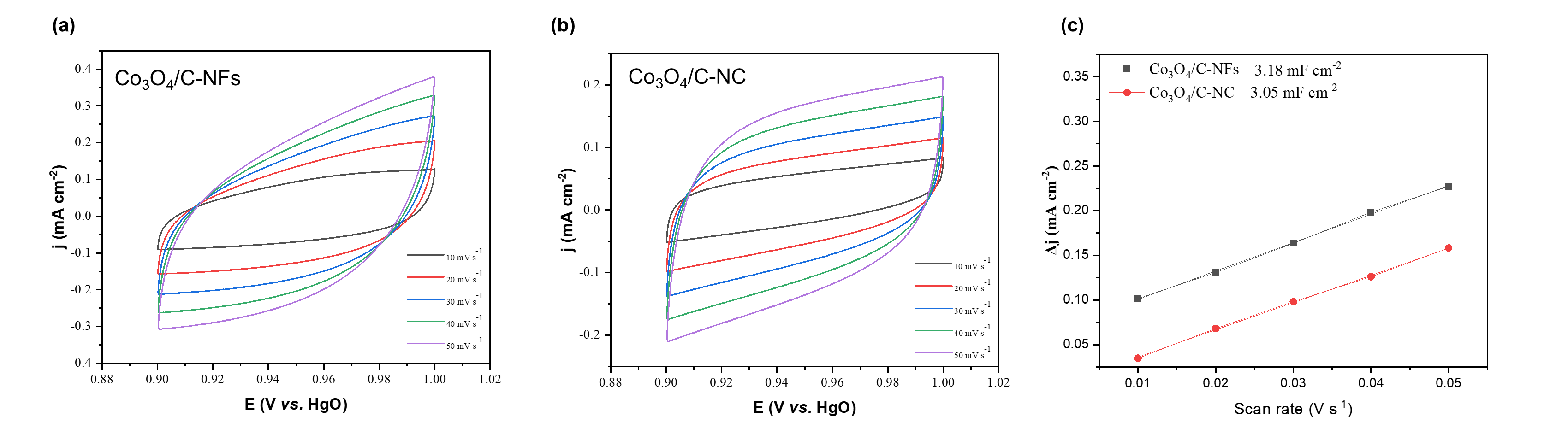


**Figure S14** CV curves of Co_3_O_4_/C-NFs (a), Co_3_O_4_/C-NC (b) at different scan rates (10, 20, 30, 40, 50 mV s^-1^). (c) Double layer capacitance fitting curves of Co_3_O_4_/C-NFs and Co_3_O_4_/C-NC.


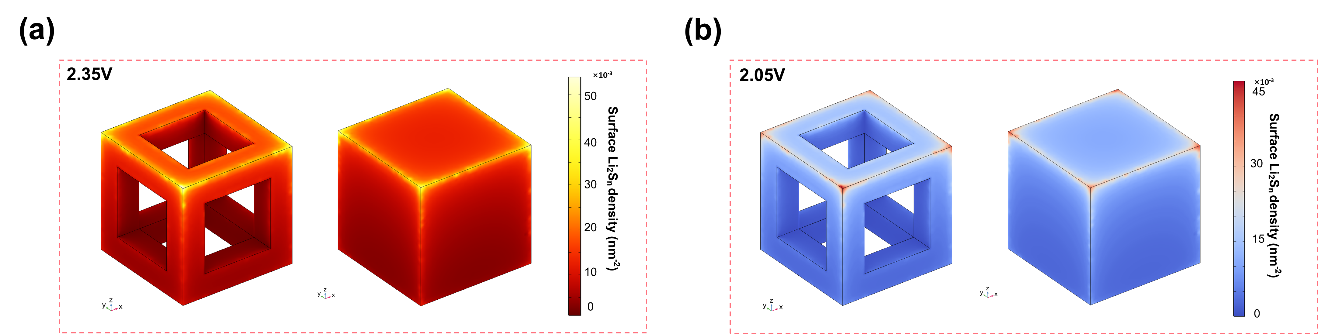


**Figure S15** The LiPSs density distribution on the surface of catalysts at 2.35 V (a), 2.05V (b).


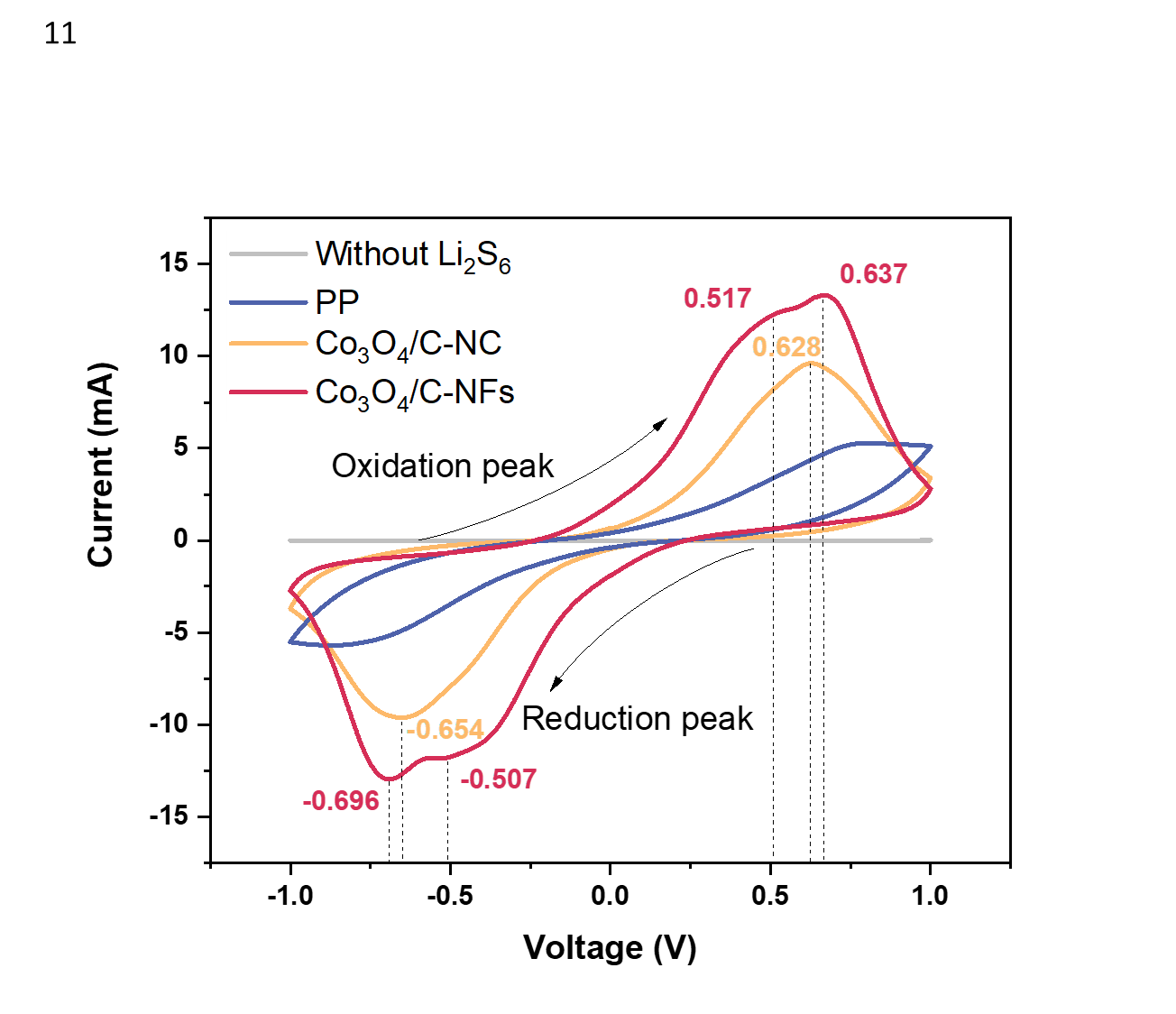


Figure S16 CV curves of Li_2_S_6_ symmetric cells assembled with different electrodes at 8 mV s^-1^.


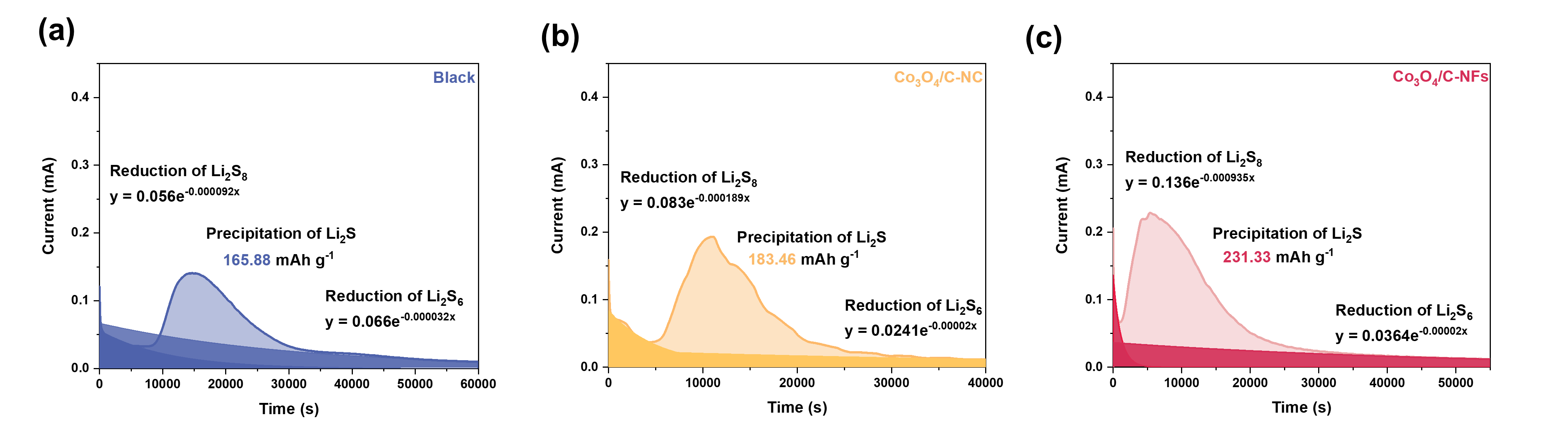


Figure S17 Potentiostatic discharge plots of cells with blank (a), Co_3_O_4_/C-NC (b) and Co_3_O_4_/C-NFs (c) electrode at 2.05 V.


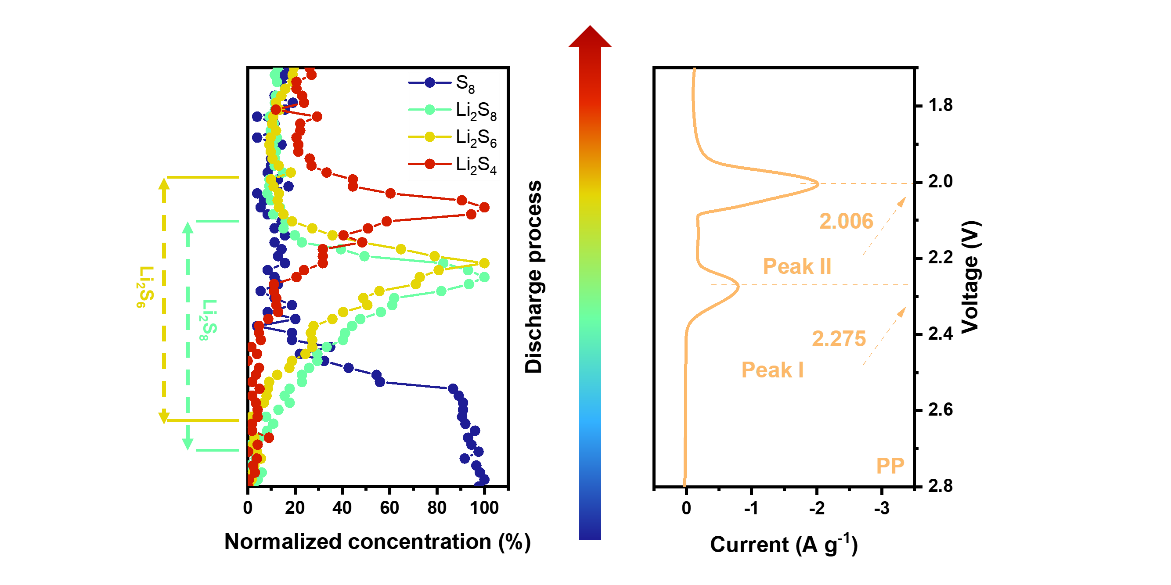


Figure S18 The content changes of different staged LiPSs and the corresponding CV curves of the Li-S batteries assembled with PP during in-situ Raman tests.


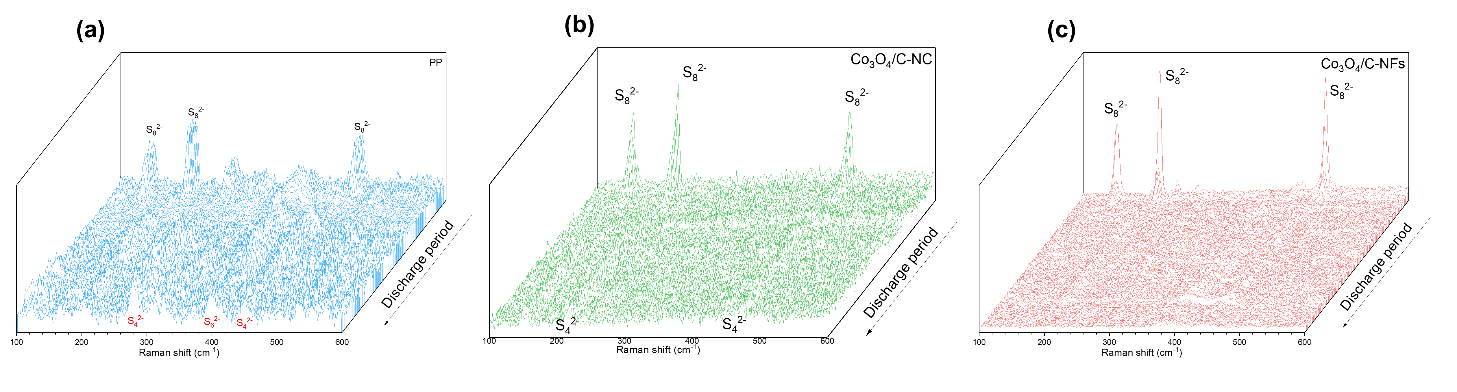


Figure S19 The origin in-suit Raman spectra of Li-S cells assembled by PP (a), Co_3_O_4_/C-NC@PP (b) and Co_3_O_4_/C-NFs@PP (c).


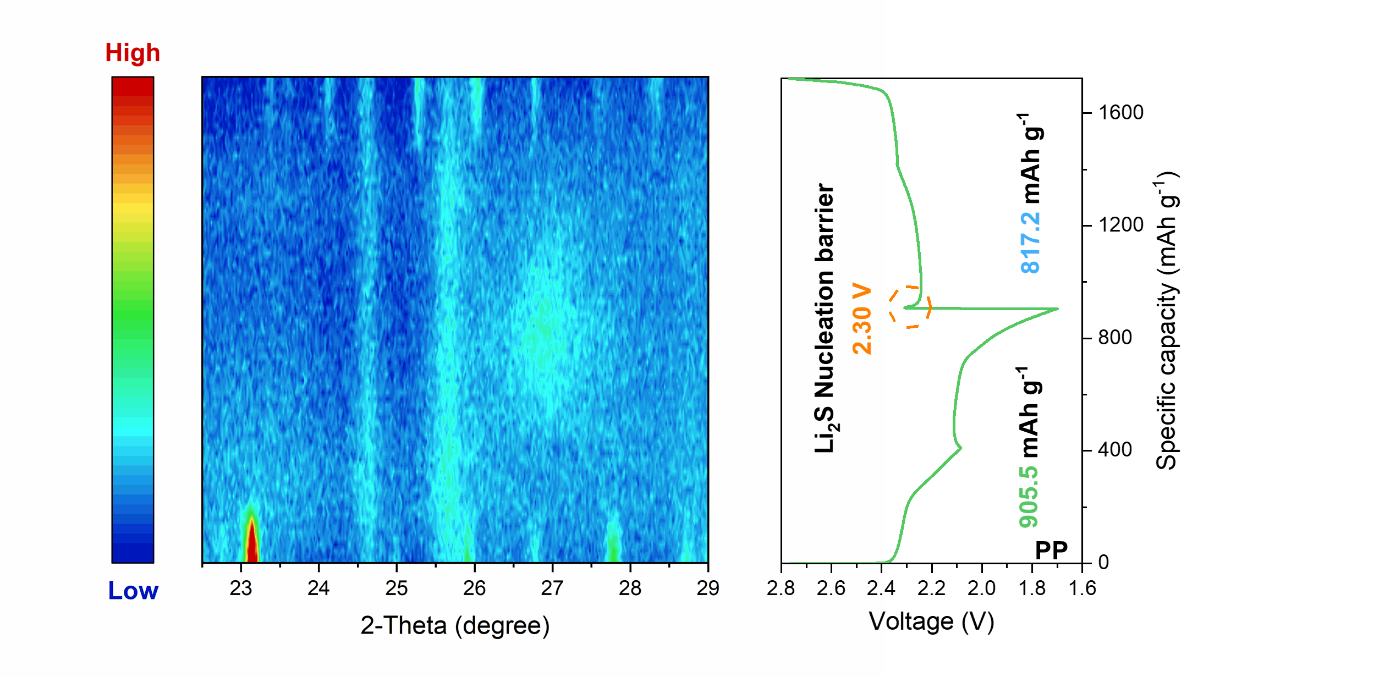


Figure S20 In-situ XRD characterization in contour plots of Li-S cells and corresponding change-discharge profiles with PP during initial cycle at 0.1 C.


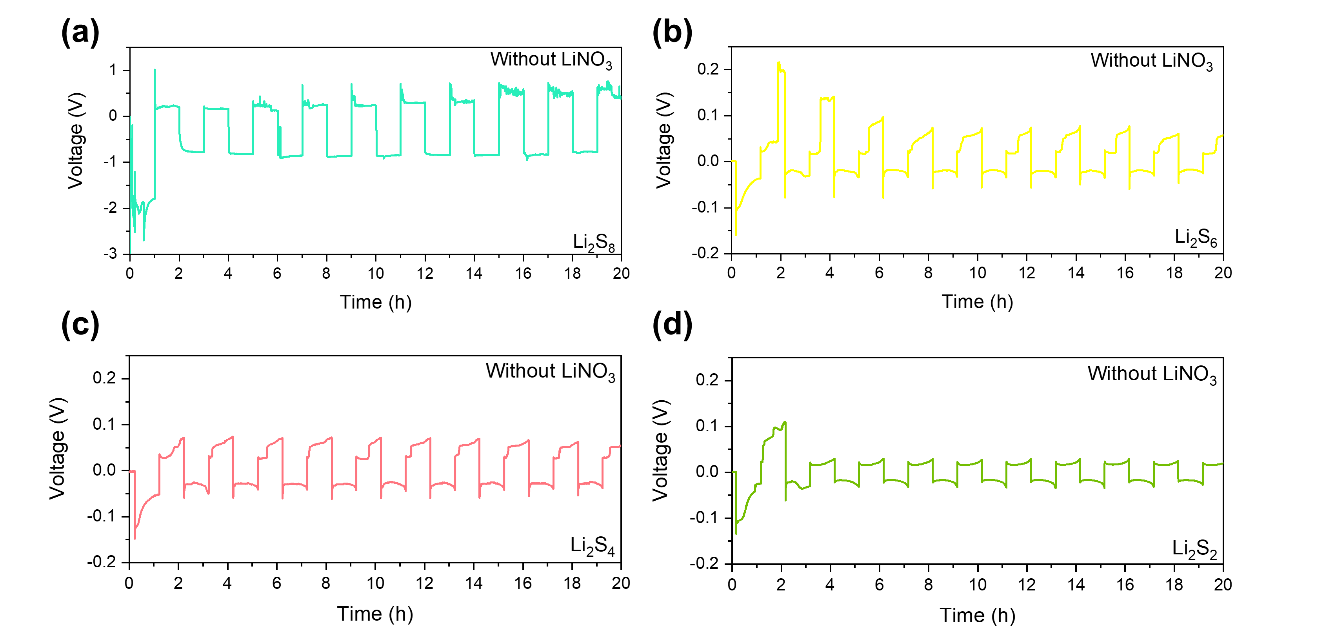


Figure S21 Cycling performance of Li-Cu cells assembled with (a) Li_2_S_8_, (b) Li_2_S_6_, (c) Li_2_S_4_ and (d) Li_2_S_2_ additives in lithium electrode side at 1 mA cm^-2^, 1 mAh cm^-2^.


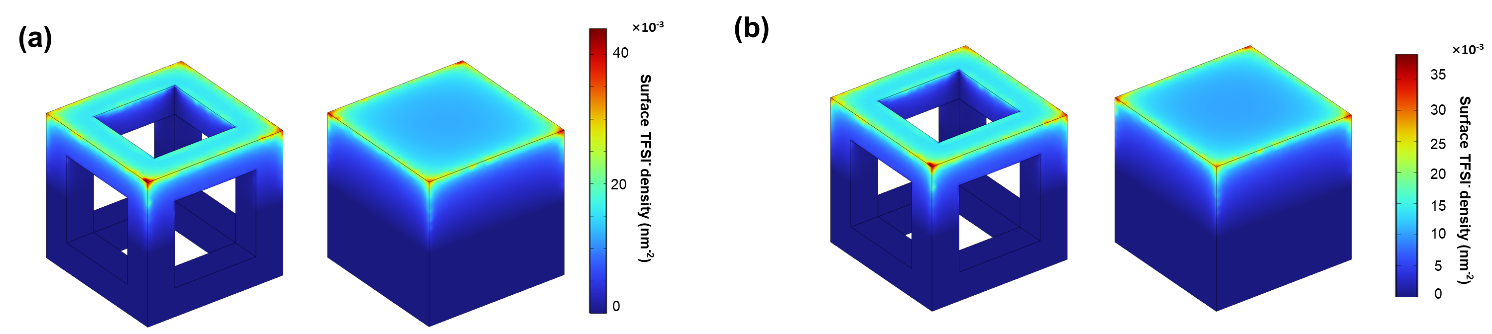


Figure S22 The TFSI^-^ density distribution on the surface of catalysts at 2.35 V (a), 2.05 V (b).


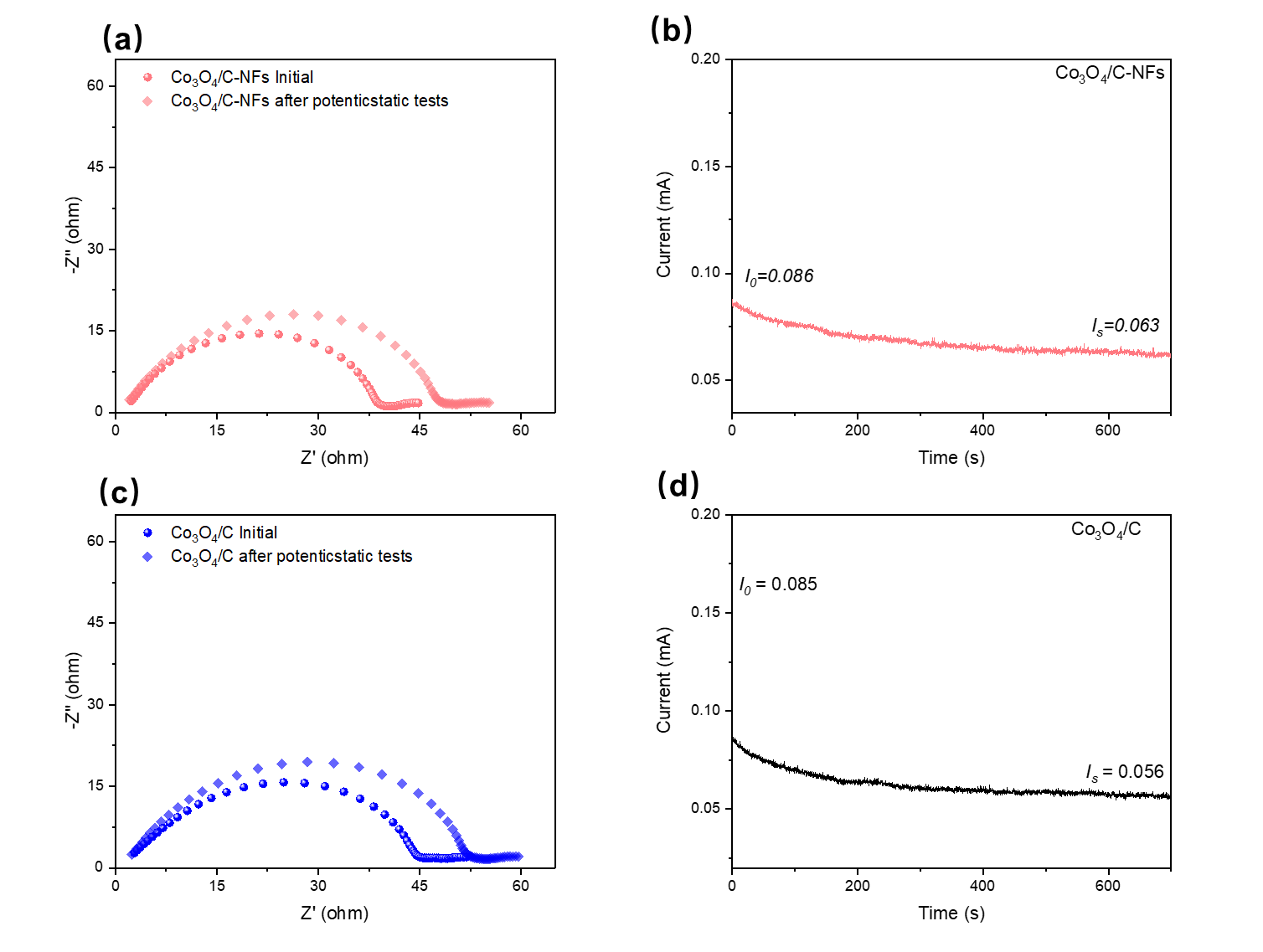


Figure S23 (a) Impedance spectra of Li|| Li symmetric cells assembled by Co_3_O_4_/C-NFs@PP before and after constant voltage testing. (b) The i-t curve of the Li-Li symmetric cells assembled by Co_3_O_4_/C-NFs@PP. (c) Impedance spectra of Li|| Li symmetric cells assembled by Co_3_O_4_/C-NC@PP before and after constant voltage testing. (d) The i-t curve of the Li-Li symmetric cells assembled by Co_3_O_4_/C-NC@PP.


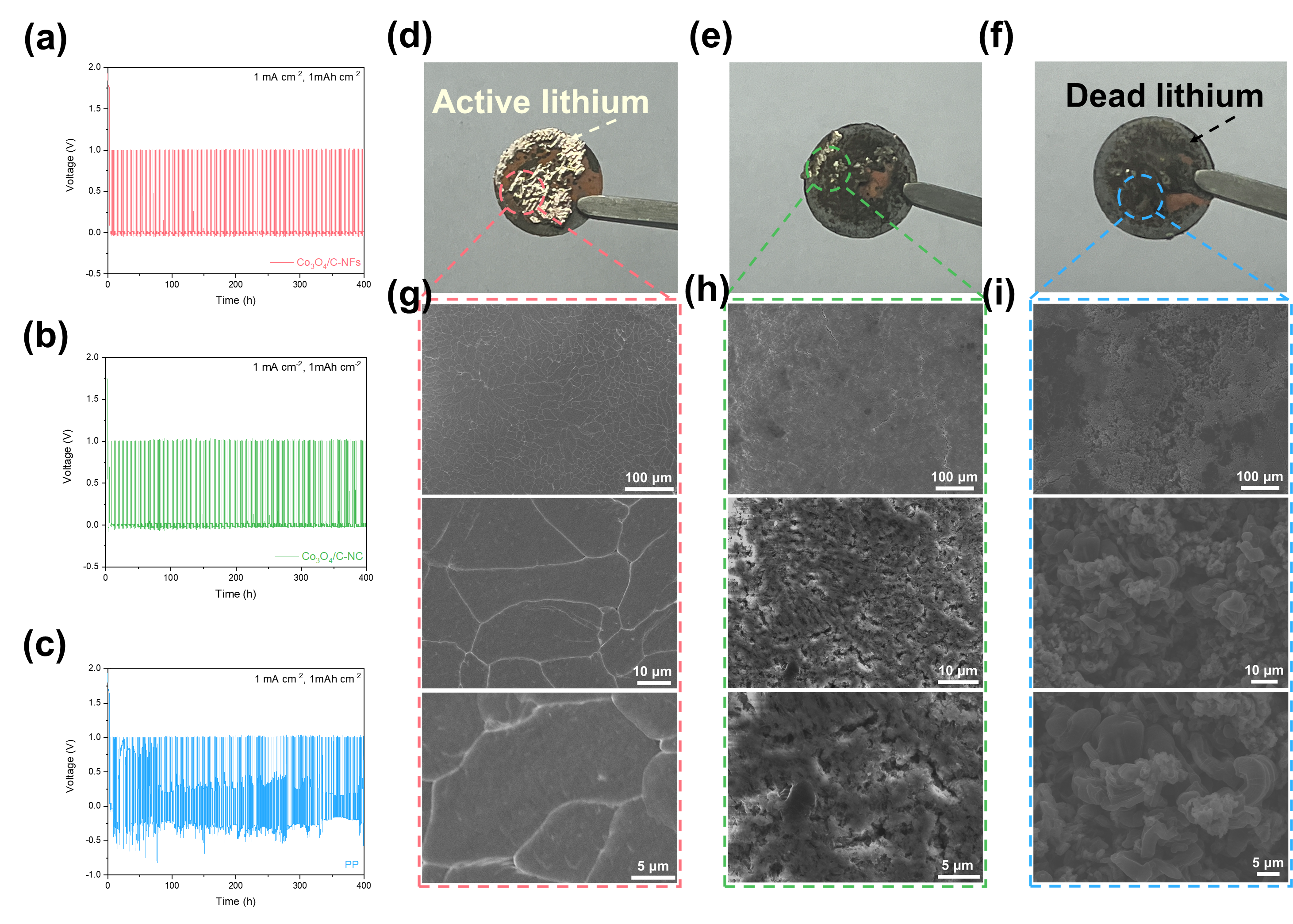


Figure S24 (a-c) The deposition voltage curves of Li-Cu cells assembled by (c) Co_3_O_4_/C-NFs@PP, (d) Co_3_O_4_/C-NC@PP and (e) PP. (f-h) The digital photos and corresponding SEM images of Cu electrode’s surface deposition morphology from Li-Cu cells assembled by (i) Co_3_O_4_/C-NFs@PP, (j) Co_3_O_4_/C-NC@PP and (k) PP after cycling tests.


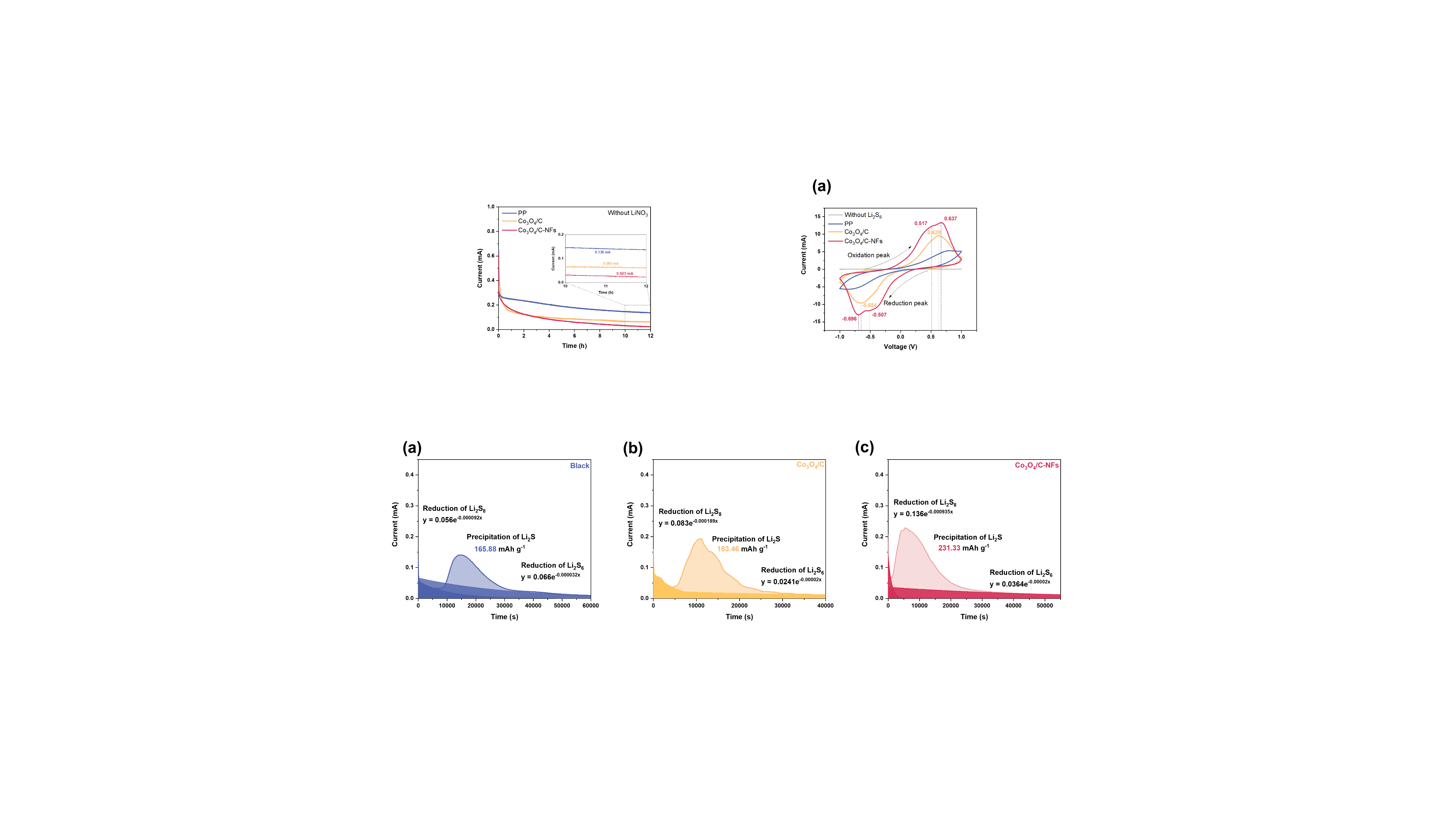


Figure S25 The shuttle currents of Li-S cells assembled with different interlayers without LiNO_3_ additive.


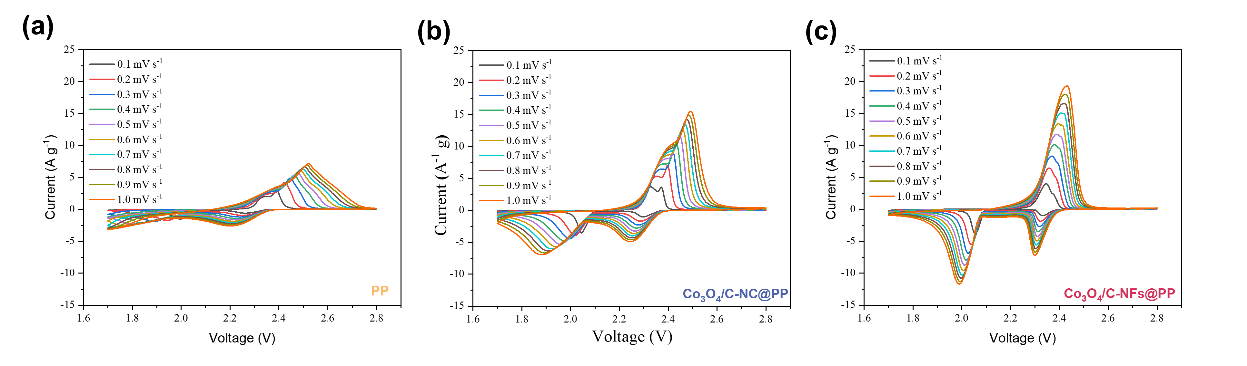


Figure S26 CV curves of Li-S cells assembled with different interlayers at different currents. (From 0.1 to 1.0 mV s^-1^).

**Table S1** Element concentrations in different samples determined by XPS analysis

|  | Co (at %) | O (at %) | C (at %) |
| --- | --- | --- | --- |
| Co_3_O_4_/C-NFs | 25.03 | 54.25 | 20.72 |
| Co_3_O_4_/C-NC | 25.15 | 53.53 | 21.32 |
